# Advancing Pain Understanding and Drug Discovery: Insights from Preclinical Models and Recent Research Findings

**DOI:** 10.3390/ph17111439

**Published:** 2024-10-28

**Authors:** Yahya I. Asiri, Sivakumar S. Moni, Mohankumar Ramar, Kumarappan Chidambaram

**Affiliations:** 1 Department of Pharmacology, College of Pharmacy, King Khalid University, Abha 62521, Saudi Arabia; yialmuawad@kku.edu.sa; 2 Health Research Centre, Jazan University, Jazan 45142, Saudi Arabia; drsmsivakumar@gmail.com; 3 Department of Pharmaceutics, College of Pharmacy, Jazan University, Jazan 45142, Saudi Arabia; 4 Department of Pharmaceutical Sciences, UConn School of Pharmacy, University of Connecticut, Storrs, CT 06269, USA; mkumar.rx@gmail.com

**Keywords:** pain, preclinical model, molecular targets, humanized models, neuroimaging, zebrafish, alternative models, *Caenorhabditis elegans*

## Abstract

Despite major advancements in our understanding of its fundamental causes, pain—both acute and chronic—remains a serious health concern. Various preclinical investigations utilizing diverse animal, cellular, and alternative models are required and frequently demanded by regulatory approval bodies to bridge the gap between the lab and the clinic. Investigating naturally occurring painful disorders can speed up medication development at the preclinical and clinical levels by illuminating molecular pathways. A wide range of animal models related to pain have been developed to elucidate pathophysiological mechanisms and aid in identifying novel targets for treatment. Pain sometimes drugs fail clinically, causing high translational costs due to poor selection and the use of preclinical tools and reporting. To improve the study of pain in a clinical context, researchers have been creating innovative models over the past few decades that better represent pathological pain conditions. In this paper, we provide a summary of traditional animal models, including rodents, cellular models, human volunteers, and alternative models, as well as the specific characteristics of pain diseases they model. However, a more rigorous approach to preclinical research and cutting-edge analgesic technologies may be necessary to successfully create novel analgesics. The research highlights from this review emphasize new opportunities to develop research that includes animals and non-animals using proven methods pertinent to comprehending and treating human suffering. This review highlights the value of using a variety of modern pain models in animals before human trials. These models can help us understand the different mechanisms behind various pain types. This will ultimately lead to the development of more effective pain medications.

## 1. Introduction

Pain is a complex physiological and sensory experience resulting from the detection of noxious stimuli by specialized receptors in the body, called nociceptors [1]. This section explores the multifaceted nature of pain by examining the complex interactions between nociception, brain processing, emotional influences, and contextual factors. Pain serves as a crucial defense mechanism, a biological alarm that sounds when tissues are at risk of injury [2]. However, severe or chronic pain can significantly impair a person’s well-being and cause both physical and psychological anguish. For the advancement of efficient interventions and treatments, identifying the etiology of pain is essential. Numerous factors, such as genetics, environmental conditions, and individual predisposition, affect the pathophysiology of chronic pain. Recent research has clarified the importance of neuroinflammation, neuronal plasticity, and alterations in neurotransmitter systems in both the initiation and long-term existence of chronic pain disorders. In addition, psychosocial factors like stress, worry, and depression can have a substantial impact on how pain is perceived and can amplify it over time [3,4].

Comprehending the etiology of pain’s fundamental causes is crucial to developing novel therapeutic techniques. Recent advances in neuroscience and molecular biology have provided insights into the molecular mechanisms behind pain signaling and have also resulted in the discovery of promising new targets for potential pain treatments [5]. Targeted pharmaceutical therapies are used in concert with non-pharmacological interventions like physical therapy and cognitive-behavioral therapy to alter pain pathways and improve pain management. Maladaptive alterations in these systems, driven by genetic, environmental, and psychosocial variables, are the cause of chronic pain syndromes. Research developments in the field of pain offer optimism regarding the creation of more efficient therapies, which will ultimately enhance the quality of life for people who experience chronic pain [6]. Traditional pain medications, including opioids, NSAIDs, antidepressants, and antiepileptics, have limitations concerning both their efficacy and safety. To develop more targeted and effective pain therapies, it is essential to discover new molecular targets. There are several promising molecular targets, such as ion channels, neurotrophins, receptors, mediators, and epigenetic modifications, which have a large amount of potential for use in future pain treatment techniques. However, the use of appropriate animal models offers a useful platform for researching pain pathways, assessing prospective medication candidates, and learning more about the underlying systems that affect how we perceive pain [7,8]. Using certain animal models, researchers can look into novel pain targets and pathways that can be changed for therapeutic purposes. The roles of certain receptors, ion channels, neurotransmitters, and signaling pathways in pain regulation by genetic modification or pharmaceutical intervention can be better understood through investigations in animals. The findings of these investigations could lead to the identification of brand-new therapeutic targets for the management of pain. The biology of pain and its related conditions have been greatly elucidated due to recent progress in animal models of pain, providing us with an improved understanding of these phenomena. Animal models continue to be an essential tool in expanding our scientific knowledge of pain and producing efficient pain-relieving drugs, even though ethical constraints and the requirement for translatability to human situations remain critical factors [9,10,11]. These models will be extremely important in expanding our capacity to lessen suffering and enhance the quality of life of those who are experiencing pain as technology and ethical issues continue to transform the landscape of pain research. This article offers a thorough review of the different pain models that are frequently used to study several pain conditions and delves into the biology at play in these models to examine how closely they resemble human pain conditions. This provides important new information about the pain signaling pathways and potential therapeutic targets. This review article also examines the benefits and drawbacks of various animal models, emphasizing the significance of studies on these models in the discovery of new analgesic medications.

## 2. Signaling Mechanism of Pain

**Figure 1:**
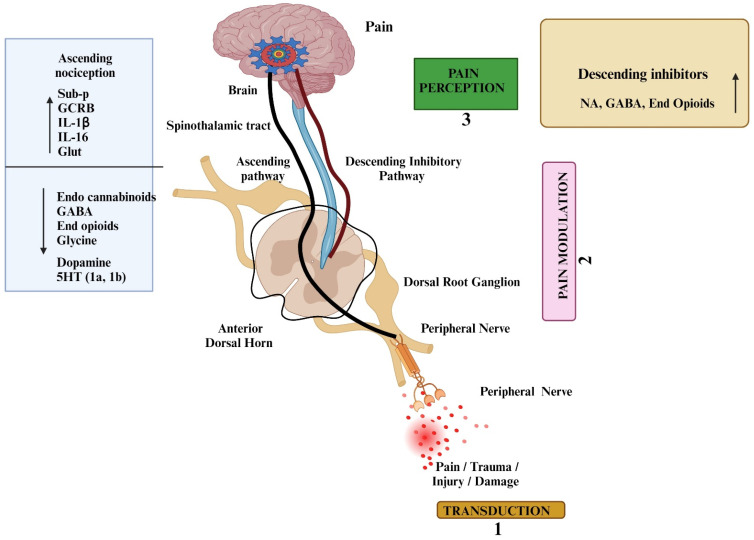
This diagram depicts the pain pathway, starting with the detection of harmful stimuli by nociceptors and ending with the perception of pain in the brain. The pathways explain the process of pain transmission including signal transduction (1), pain modulation (2), and pain perception by the brain. Ascending involves the release of pain mediators (Sub-P—Substance P; Glut—glutamate; CGRP—calcitonin gene-related peptide; IL-interleukins (1β, 16); and pain modulators (endogenous cannabinoids, GABA—gaba amino butyric acid; endogenous opioids, glycine, dopamine, and 5HT-5 hydroxy tryptamine). Descending pathways inhibit the transmission through various mediators (NA-norepinephrine; GABA; and Endogenous opioids). 1—Pain Tranduction refers to The mechanisms by which tissue damage triggers the activation of nerve endings involve a complex series of events; 2—Pain Modulation refers to the process of altering pain signals as they travel along the pain pathway; 3—Pain Perception is an unpleasant sensation, both physical and emotional, that arises from actual or potential tissue injury; ↑ increase; ↓ decrease. All images in this figure were created with BioRender.com, BioRender, Canada (BioRender.com).

Nociception refers to the sensing of intense stimuli by specialized peripheral nerve fibers called nociceptors. These nociceptors are selectively triggered when the stimuli surpass a certain threshold. Notably, nociceptors exhibit diversity in their neurotransmitter composition, expression of receptors, and sensitivity to noxious stimuli. The basic pathways of pain transmission and its associated mechanism are illustrated in Figure 1. These specialized nerve endings can directly detect and respond to detrimental stimuli. Additionally, they can be indirectly activated through the release of chemicals from injured tissues, such as bradykinin, substance P, and various peptides. Sensory transmission occurs through axons that are either unmyelinated or possess a thin myelin sheath and small diameter [12,13]. The type Aδ fibers are involved in transmitting intense, brief mechanical stimuli. Within the type Aδ fiber group, there are two subtypes: type I and type II. Type I Aδ fibers are sensitive to high temperatures (thresholds above 50 °C), while type II Aδ fibers have a higher tolerance for mechanical stimuli but respond to lower temperatures. Notably, type II Aδ fibers play a significant role in conveying thermal signals. In contrast, C-fibers transmit slow, dull signals associated with secondary pain. Nociceptors can be classified into two subgroups: peptidergic and non-peptidergic. Peptidergic nociceptors express receptors for neuropeptides such as Substance P and calcitonin gene-related peptide (CGRP). Recent research has unveiled the presence of transient receptor potential (TRP) channels in nerve endings, which play a critical role in detecting and responding to cellular damage. These TRP channels exhibit similarities to voltage-gated potassium channels and nucleotide-gated channels [14].

The TRP (transient receptor potential) receptor family includes a subclass known as thermal nociceptive receptors. Within this subclass, the transmission of heat stimuli is mediated by the vanilloid-type (TRPV) channels, which consist of several ligand-specific subfamilies. TRPV channels are expressed in thermal nociceptors. The TRPV channels are composed of four subunits, which form tetrameric structures with central channels in the cell membrane. These tetramers can be either homo- or hetero-tetrameric in composition. Among them, TRPV1 and TRPV2 are key receptors responsible for detecting and responding to high temperatures that can potentially cause damage (noxious stimuli). TRPV1 is predominantly located on heat-sensitive C-fibers, while TRPV2 is found on A-delta fibers, another type of heat-sensitive nerve fiber. TRPV1 is activated at temperatures around 40–43 °C, whereas TRPV2 requires a higher temperature of approximately 52 °C for activation [15,16].

Recent studies have shown that TRPV1 is distributed across multiple areas of the central nervous system. TRPV1 and TRPV2 open to enable calcium to enter and depolarize the neuron when they sense a temperature that is high enough. At negative holding potentials, the activation of TRPV1 leads to an influx of calcium and sodium ions, depolarizing the cell. TRPV2, located in the Aδ and Aβ nerve fibers, is involved in transmitting sensations of intense heat and detecting harmful chemicals. This indicates that TRPV2 plays a role in nociception, the process of perceiving pain. Both the TRPV1 and TRPV2 channels are non-selective cation channels with a preference for sodium or calcium. They trigger a cascade of events that lead to the transmission of pain signals, making them potential targets for pain management. An afferent action potential is produced when adjacent voltage-gated sodium channels are stimulated by calcium-induced depolarization [17,18,19]. TRPA1 outperforms TRPV1 in terms of chemical sensitivity. ‘A’ denotes subfamily A, whereas ‘V’ denotes vanilloid. Capsaicin, prostaglandins, bradykinin, and other significant pro-inflammatory molecules can be bound by TRPV1, but TRPA1 can detect a wide array of oily and pungent isothiocyanate compounds, including formaldehyde, mustard oils, and cinnamonaldehyde, which is frequently injected to investigate the activity of TRPA1 receptors. TRPV3 and TRPV4, the other two components of the TRPV receptor family, exhibit responsiveness to a broader range of warmth. TRPV3 is activated within the temperature range of 27–34 °C, whereas TRPV4 activation occurs between 33 and 39 °C. Collectively, these TRPV receptors enable the detection of a broad spectrum of thermal sensations, spanning from mildly discomforting to potentially harmful temperatures, and including those within the normal physiological range [18].

## 3. Epigenetic Pathway of Pain

Epigenetics involves the study of heritable changes in gene expression that do not involve alterations in the underlying DNA sequence. These changes, called epigenetic modifications, can influence the expression of genes related to pain, including those involved in detecting pain signals and transmitting them to the brain. The epigenetic route to pain is a regulatory network wherein epigenetic modifications are used to modify gene expressions. The epigenetic pathway of pain can be characterized by several mechanisms. Epigenetic alterations have the potential to impact the expression of nociception-related genes, thus influencing pain perception and sensitization [20]. When looking at nociceptors and peripheral sensitization, variations in DNA methylation can affect the expression of genes which regulate nociceptor activity. These findings have implications for individuals’ pain sensitivity and responses to inflammation signals [21,22]. This epigenetic process can regulate genes that encode receptors, ion channels, and other elements critical for detecting and transmitting pain signals. Shifts in methylation patterns can alter these components’ expression and thus affect the nociceptors’ sensitivity to painful stimuli [23]. Methylation shifts within genes related to immune activities and inflammation can modify the body’s reaction to harm or trauma [24,25]. Epigenetic changes in these genes can affect neurotransmitter levels and signaling pathways, influencing how pain signals are transmitted and processed. DNA methylation can create an “epigenetic memory” of pain experiences. This means that past painful experiences could lead to lasting changes in the epigenetic landscapes of relevant genes, potentially contributing to the formation of chronic pain conditions [26]. Hypermethylation often leads to gene silencing, while hypomethylation can result in gene activation [27]. Aberrant DNA methylation patterns have been associated with chronic pain conditions. For instance, hypermethylation of the OPRM1 gene, which encodes the mu-opioid receptor, can reduce opioid efficacy. Further, recent studies have demonstrated that the hypermethylation of certain genes involved in pain pathways, such as BDNF (brain-derived neurotrophic factor) and NR1 (NMDA receptor subunit 1), can contribute to chronic pain. Several other studies have revealed that epigenetic mechanisms, including histone acetylation, non-coding RNAs, and DNA methylation, can influence the expression of BDNF. These epigenetic modifications may contribute to the pathogenesis and symptomatology of chronic pain [28,29]. A recently published study reviewed the epigenetic modifications involved in the transition from acute to chronic pain. The researchers identified DNA methylation and histone modifications as critical factors in this process, suggesting that targeting these pathways could prevent chronic pain development [30]. These changes can significantly impact various biological processes, including the development and persistence of pain. Animal studies are crucial for understanding the genetic factors that contribute to chronic postsurgical pain (CPSP). Mutations in the Cacng2, p2rx7, and BDNF genes have been linked to CPSP susceptibility. DNA methylation and microRNAs are involved in gene regulation and may both have therapeutic potential. In children, changes in DNA methylation patterns point to shared mechanisms related to GABAergic, dopaminergic, and immune functions [31].

Histone modifications, including acetylation, methylation, phosphorylation, and deacetylation, play crucial roles in regulating gene expressions related to pain perception and sensitization [32]. Histone acetylation generally promotes gene expression, while deacetylation represses it. Inhibitors of histone deacetylases (HDACs) have shown promise in reducing pain by enhancing the expression of anti-inflammatory genes. For example, methylation at specific sites can increase the expression of genes related to pain signaling, while methylation at other locations can decrease their expression [33]. The inhibition of histone deacetylases has been investigated as a potential approach to mitigating chronic pain by modulating the expression of genes associated with pain. These modifications impact the chromatin structure and gene accessibility, contributing to pain pathways and offering potential therapeutic targets [27].

MicroRNAs (miRNAs) and long non-coding RNAs (lncRNAs), which belong to the category of non-coding RNAs (ncRNAs), are involved in the regulation of gene expression and the cellular mechanisms associated with pain perception [34,35]. MiRNAs inhibit translation or degrade messenger RNAs (mRNAs), influencing nociceptor function and pain signaling pathways. These specific miRNAs have been implicated in pain modulation. For example, miR-124 is known to suppress inflammatory responses and is downregulated in chronic pain conditions [36]. The aberrant expression of microRNAs (miRNAs) is associated with chronic pain disorders, as they lead to changes in pain perception. LncRNAs interact with DNA, RNA, and proteins, regulating gene expression and cellular pathways involved in both pain conditions [37]. They modulate genes related to inflammation, neurotransmission, and immune responses, impacting pain perception and sensitization. Understanding these epigenetic mechanisms provides insights into pain management strategies.

## 4. Mediators and Molecular Targets of Pain—An Overview

**Figure 2:**
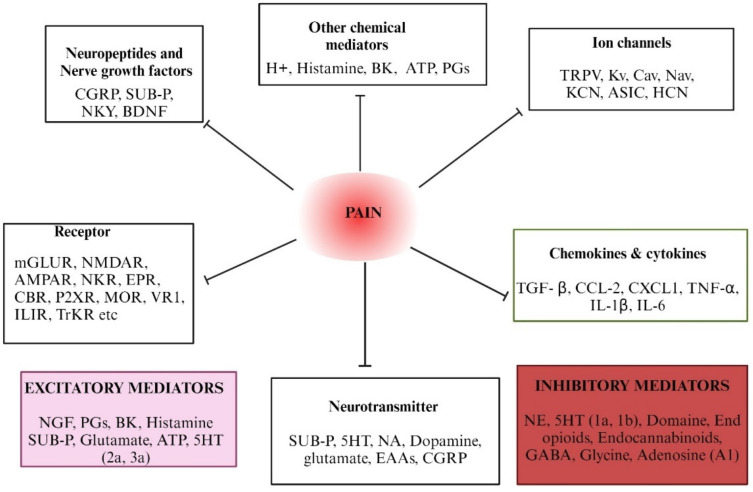
The types of neurochemicals (including neurotransmitters and neuropeptides), ion channels, and receptors involved in the pain process. The excitatory and inhibitory mediators are depicted in the illustration. Following, nerve damage, various receptors and ion channels are activated by excitatory neurochemicals and further sensitized by cytokines. Neuropeptides and nerve growth factors (NGF): CGRB—calcitonin gene-related peptide; Sub-P—substance P; NKY—neurokinin Y; BDNF—brain-derived neurotrophic factor; other chemicals: H+, ATP—adenosine triphosphate; PGs—prostaglandins; BK—bradykinin; ion channels: TRPV—transient receptor potential vanilloid; Kv—voltage-gated potassium channel; Nav—voltage-gated sodium channel; Cav—voltage-gated calcium channel; KCN—potassium channels; ASIC—acid-sensing ion channels; HCN—hyperpolarization-activated cyclic nucleotide-gated channels; chemokines and cytokines: TGF-β1—transforming growth factor beta 1; CCL2—C-C motif chemokine ligand 2; CXCL1—chemokine (C-X-C motif) ligand 1; TNF-α—tumour necrosis factor alpha; IL-1β—interleukin 1β; IL6—interleukin 6; receptors: mGLUT—metabotropic glutamate receptors; NMDAR—N-methyl-D-aspartate receptor; AMPAR—α-amino-3-hydroxy-5-methylisoxazole-4-propionic acid receptor; NKR—neurokinin receptor; EPR—E type-prostaglandin receptor; CBR—cannabinoid receptor; P2XR- ionotropic purinergic receptors; MOR—mu-opioid receptors; VR1—vanilloid receptor 1; IL1R—interleukin 1 receptor; TRKR—tyrosine kinase receptor; neurotransmitters: 5HT-5 hydroxy tryptamine; NA—noradrenaline; EAAs—excitatory amino acids. All images in this figure were created with BioRender.com, Bio Render, Canada (BioRender.com).

Pain mediators play a crucial role in initiating, propagating, and modulating pain signals. Understanding the molecular targets and mediators involved in pain pathways is crucial for developing effective analgesic drugs. Several key molecular targets and mediators of pain, representing recent advances and their implications for pain drug discovery, have been discussed in various review articles [38,39,40]. Pro-inflammatory cytokines, such as TNF-α, IL-1β, and IL-6, and chemokines (e.g., CCL2) are released in response to tissue injury and inflammation. Figure 2 illustrates the various neurochemicals and transmitters, as well as the role of ion channels, related to the mechanism of pain. These cytokines contribute to both peripheral and central sensitization, leading to chronic pain conditions. They activate nociceptors and enhance pain sensitivity through the upregulation of pain-related receptors and ion channels [41]. Chemokines like CCL2 and CXCL1 attract immune cells to the site of injury, promoting inflammation and pain. They interact with the receptors on nociceptors, leading to increased pain sensitivity. These mediators are released by immune cells and act on their respective receptors on nociceptors. Chemokine signaling pathways play a crucial role in maintaining chronic pain by sustaining inflammatory responses. Inhibiting these pathways can alleviate pain by reducing inflammation and immune cell recruitment. Targeting pro-inflammatory cytokines and chemokines with their specific inhibitors can reduce pain and inflammation, offering a promising approach for pain management.

Prostaglandin E2 (PGE2) is another lipid mediator that sensitizes nociceptors by binding to EP receptors. It is involved in the sensitization of the TRPV1 receptor, which is crucial for inflammatory pain. This sensitization occurs predominantly through a PKC-dependent pathway. Particularly, EP1 receptors contribute to thermal hyperalgesia and inflammatory nociceptive responses. Recent experiments have shown that mice lacking TRPV1 or EP1 receptors experience less pain from heat and inflammation. This suggests that PGE2 or PGI2 may increase pain sensitivity by activating TRPV1 receptors through EP1 or IP receptors [42]. The complexity of EP receptor signaling, and their involvement in various physiological processes, can make it challenging to develop selective drugs without off-target effects. Understanding the roles of EP receptors can lead to targeted therapies for pain management, reducing the side effects associated with non-specific treatments. Sensory nerves contain and release a variety of neuropeptides, which differ in their chemical composition. Neuropeptides like Substance P and calcitonin gene-related peptide (CGRP) are released from sensory neurons and play a crucial role in pain transmission and modulation [12,43]. They enhance pain signaling by increasing the excitability of nociceptors and promoting neurogenic inflammation. Antagonists that target neuropeptide receptors have demonstrated efficacy in reducing pain in various preclinical and clinical studies. Nerve growth factor (NGF) plays a crucial role in the development, maintenance, and function of sensory neurons, including those involved in pain perception. NGF may also contribute to central sensitization, a condition in which the central nervous system becomes hypersensitive to pain signals. NGF binds to the TrkA receptor, a tyrosine kinase receptor expressed on the surface of sensory neurons, which leads to the autophosphorylation of the receptor, which in activates downstream signaling cascades, including the Ras-MAPK pathway and the PI3K-Akt pathway. Drugs that interfere with the Ras-MAPK or PI3K-Akt pathways are able to reduce NGF-mediated effects [44]. Future research will continue to investigate the role of NGF and its signaling mechanisms in pain, exploring innovative therapeutic approaches and addressing the challenges associated with targeting this pathway.

Importantly, ion channels, such as voltage-gated sodium channels (Nav1.7, Nav1.8) and transient receptor potential (TRP) channels, play a key role in the generation and transmission of pain signals. Studies have shown that disruptions in ion channel function can contribute to abnormal pain perception and chronic pain conditions [45]. Targeting ion channels with specific blockers or modulators can offer effective pain relief. Additionally, endocannabinoids like anandamide and 2-arachidonoylglycerol (2-AG) can modulate pain by activating cannabinoid receptors (CB1 and CB2) in the nervous system. These mediators help to inhibit pain transmission and reduce inflammation [46]. Furthermore, glial cells, including microglia and astrocytes, release pro-inflammatory mediators that can enhance pain signaling in the central nervous system. Targeting the activation of glial cells and their mediators may be beneficial in reducing central sensitization and chronic pain. Understanding these pain mediators is essential for developing effective pain management strategies. Accordingly, targeting specific molecules or pathways, researchers and clinicians can develop new analgesics and therapies to alleviate pain.

## 5. Classical Pain Models—An Overview

**Figure 3:**
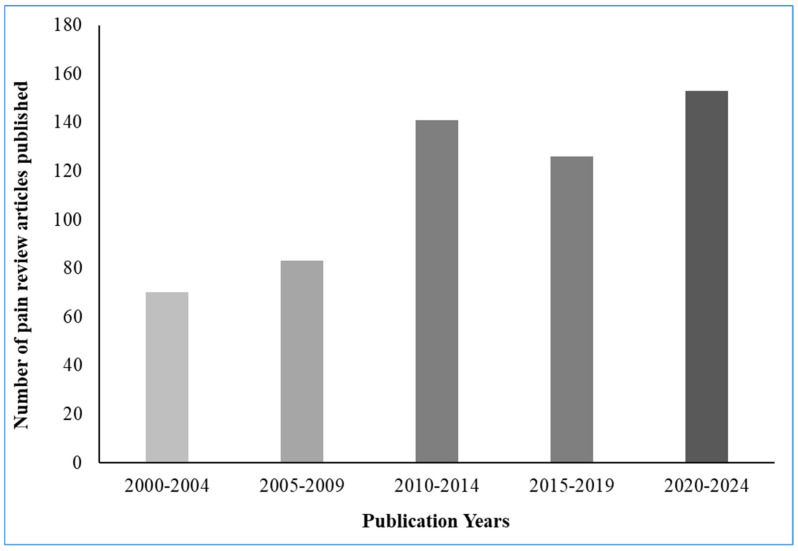
The number of review articles published on animal models of pain from 2000 to 2023. The figure shows the total number of review articles on rodent models of pain represented in the PubMed database by year of publication, identified using the MeSH search terms ‘review’, ‘animal’, ‘models’, and ‘pain’ (Source from PubMed).

By providing valuable insights into pain, conventional models have laid the groundwork for the development of even more effective pain treatments. These models involve stimulating animals with heat, pressure, or chemicals to induce pain-like responses. Common examples include the hot plate test, formalin test, and acetic acid writhing test [47,48] While they cannot perfectly replicate the full experience of human pain, these models prove helpful in understanding how pain signals travel through the body (nociceptive processing), how the body naturally regulates pain (pain modulation), and whether pain medications are working (analgesic efficacy). Despite their limitations, classical pain models remain crucial tools for understanding pain mechanisms at a fundamental level, testing potential pain medications, and evaluating treatment effectiveness. The accompanying figure (Figure 3) illustrates the quantity of pain review articles published within the entire literature spanning the years from 2000 to 2023.

### 5.1. Inflammatory Pain Models

Inflammatory pain, which arises from tissue damage and inflammation, constitutes a significant element of numerous chronic pain disorders due to its intricate nature. Studying inflammatory pain models in animals has been fundamental in understanding its underlying biological mechanisms and identifying potential therapeutic targets [49]. Researchers have heavily relied on animal models to comprehend the complex biology of pain perception and develop novel therapeutic interventions for pain management. Carrageenan-induced paw oedema, Complete Freund’s Adjuvant (CFA)-induced arthritis, and formalin-induced paw inflammation are some commonly used models to study inflammatory pain in animals. Research using this model has revealed the involvement of various inflammatory mediators, such as prostaglandins, cytokines (e.g., tumor necrosis factor-alpha, interleukins), and bradykinin, in the sensitization of nociceptive pathways [50,51]. Moreover, research has exhibited that NSAIDs effectively reduce carrageenan-induced pain, validating the model’s relevance for screening potential analgesic agents [52,53]. The primary objective of this inflammatory model is to enhance our comprehension of pain, ultimately resulting in improved pain treatments for both humans and animals.

#### 5.1.1. Complete Freund’s Adjuvant (CFA) Induced Inflammatory Pain Model

CFA, an immune adjuvant, is injected into the joints, leading to persistent inflammation and pain. CFA-induced pain is characterized by joint swelling, heat hypersensitivity, and mechanical allodynia. The CFA-induced arthritis model has shed light on the role of immune cells, including macrophages and T cells, in perpetuating inflammatory pain conditions [54]. A promising approach to treating pain in CFA-induced arthritis involves selectively targeting pro-inflammatory cytokines such as TNF-alpha and interleukins [55,56]. The early phase is attributed to the direct activation of nociceptors, while the late phase involves a release of inflammatory mediators that is caused by both acute and chronic inflammatory pain. Research findings from this model have revealed the involvement of TRP channels, such as TRPV1 and TRPA1, in mediating formalin-induced pain responses [57,58]. Moreover, studies have demonstrated the effectiveness of targeting TRP channels and blocking inflammatory mediators in attenuating formalin-induced pain, suggesting their therapeutic potential [59]. Inflammatory pain models have provided invaluable insights into the complex biological processes underlying chronic pain conditions. Studies using these models have identified key inflammatory mediators, immune cells, and molecular targets that contribute to inflammatory pain. Unlocking these mechanisms has opened doors to novel pain relievers (analgesic drugs) and personalized treatments (targeted therapies) for managing chronic pain.

#### 5.1.2. Formalin-Induced Nociceptive Pain Model

The formalin test is a widely used animal model for studying pain mechanisms and evaluating potential analgesic drugs [60]. This section discusses the mechanisms, principles, limitations, advantages, validity, and strengths of the formalin test, with a focus on its relevance to human pain and its utility in pain drug discovery. The formalin test induces a biphasic pain response characterized by two distinct phases (acute and chronic). The first phase is primarily mediated by peripheral nociceptors and the release of inflammatory mediators, such as prostaglandins and bradykinin. These mediators sensitize the nociceptors, increasing their responsiveness to noxious stimuli. The second phase is thought to involve central sensitization, a process whereby the spinal cord and brain become hyperexcited, leading to amplified pain signals [61]. Further, one study indicated that formalin stimulates sensory neurons by directly activating TRPA1, a cation channel essential for the development of inflammatory pain [57]. Pain behaviors such as licking and flinching are easily observable and quantifiable, facilitating the objective assessment of analgesic efficacy. In addition, the formalin test is highly reproducible across different laboratories, making it a reliable model for pain research. The formalin test has been pivotal in advancing our understanding of pain mechanisms and identifying potential therapeutic targets. Despite species differences and other limitations, the model’s ability to replicate key features of human inflammatory pain makes it a valuable tool in preclinical pain research [62]. The insights gained from this model have led to the development of novel analgesics that have shown efficacy in clinical trials [63]. Recent research has continued to refine the formalin test and to investigate its relevance to human pain. For example, studies have explored the roles of different nociceptor subtypes in the formalin response and have investigated the effects of various pharmacological agents on the test [64]. The formalin test remains a cornerstone in pain research, offering a robust and reproducible model for studying pain mechanisms and evaluating analgesic drugs. While it has limitations, its strengths and validity make it an indispensable tool in the quest to understand and alleviate human pain. Continued research is needed to further refine the test and to explore its relevance to human pain conditions.

### 5.2. Visceral Pain Mechanisms Through Animal Models

#### The Colorectal Distension (CRD) Model

The colorectal distension (CRD) model is a widely employed method to study visceral pain, and is conducted by inserting a balloon catheter into the colorectal region, after which distension is applied to induce the visceral pain associated with irritable bowel syndrome [65]. Various neurotransmitters, such as CGRP and 5-HT, have been identified as mediating visceral pain sensitization [66]. Visceral inflammation and pain responses are linked to another type of visceral pain that is induced by acetic acid. This model has highlighted the role of TRP channels, such as TRPV1 and TRPA1, in mediating visceral pain sensitization [67]. Moreover, studies have demonstrated the effectiveness of targeting TRP channels and blocking inflammatory mediators in attenuating acetic acid-induced visceral pain. Involving the employment of a chemotherapeutic agent, the cyclophosphamide-induced cystitis pain model is specifically used to study visceral pain related to bladder inflammation, pelvic allodynia, and cystitis. This chemical-induced-pain model has revealed the role of purinergic signaling, particularly the activation of the P2X3 receptors, in mediating visceral pain [68]. Moreover, studies have demonstrated the effectiveness of targeting the P2X3 receptors and blocking purinergic signaling in alleviating cyclophosphamide-induced visceral pain [69,70]. The use of visceral pain models has been instrumental in advancing our understanding of the complex mechanisms involved in chronic visceral pain conditions. These models have provided valuable insights into the underlying mechanisms of such conditions. These ongoing efforts hold promise for better pain control and improved quality of life in patients with visceral pain conditions.

### 5.3. Mechanisms and Models of Neuropathic Pain

Neuropathic pain is a debilitating and challenging condition resulting from nerve damage or dysfunction, and is often associated with trauma, infection, or diseases like diabetes and multiple sclerosis. Identifying the mechanisms involved in neuropathic pain is essential for creating successful treatment strategies. Unlocking the mechanisms behind neuropathic pain holds the key to creating better treatments for this challenging condition [71,72]. Neuropathic pain models, frequently employing nerve injury techniques, successfully replicate different facets of chronic pain observed in humans, enabling researchers to investigate the intricate mechanisms involved. This has resulted in notable progress in the development of pain medications. Nonetheless, a significant limitation arises from the inherent disparities in nervous system physiology between animals and humans. Recent advancements are focused on enhancing existing models by incorporating more sophisticated pain behaviors that better align with the human experience. Moreover, scientists are exploring alternative models that utilize genetic modifications to simulate specific pain disorders. Despite these constraints, they continue to be a valuable tool, and ongoing refinements offer promising prospects for even more precise neuropathic pain research in the future.

#### 5.3.1. The Chronic Constriction Injury (CCI)

The chronic constriction injury (CCI) method is a widely utilized experimental animal model for neuropathic pain. It involves loosely ligating a section of the sciatic nerve, which induces partial nerve constriction and subsequently gives rise to neuropathic pain behavior. Studies conducted using this model have unveiled the involvement of sodium channels, specifically Nav1.7 and Nav1.3, in the mediation of neuropathic pain [73]. Moreover, studies have indicated that suppressing inflammatory cytokines and chemokines, such as TNF-α and IL-6, can offer pain relief from CCI-induced discomfort. This suggests that these molecules hold potential as viable targets for therapeutic intervention [74,75]. While the CCI model has been invaluable for studying neuropathic pain, it has certain limitations. For example, it primarily models peripheral neuropathic pain and may not fully capture the complexities of central neuropathic pain. Additionally, the degree of nerve compression attained using the CCI model varies significantly across studies, leading to inconsistencies in research findings and hindering progress towards understanding neuropathic pain. Future research efforts should focus on refining the CCI model and developing new models to better represent different types of neuropathic pain.

#### 5.3.2. Spinal Nerve Ligation Model

Spinal nerve ligation (SNL) exhibits notable features of mechanical allodynia (heightened sensitivity to gentle touch) and thermal hyperalgesia (heightened sensitivity to heat). Research conducted using this model has provided significant knowledge regarding the participation of NMDA receptors and BDNF in the process of central sensitization, which is a fundamental mechanism in the development of persistent pain. These findings have contributed to a deeper understanding of the mechanisms underlying persistent pain conditions [76,77]. Additionally, research has shown that targeting glial cells, specifically microglia and astrocytes, can effectively reduce neuropathic pain induced by spinal nerve ligation (SNL). These studies have provided evidence for the potential therapeutic value of modulating glial cell activity, as it can alleviate neuropathic pain [78,79,80]. The ligation results in long-lasting behavioral signs of mechanical allodynia, heat hyperalgesia, cold allodynia, and ongoing pain. By studying this type of nerve pain, researchers can learn more about how pain works after nerve damage is sustained. This knowledge helps them to develop and test new medications to effectively manage pain [81].

#### 5.3.3. Chronic Constriction of the Infraorbital Nerve Model

The CCI-ION model is a specific experimental model used to examine neuropathic pain resulting from trigeminal nerve injuries. It allows researchers to explore the mechanisms and features of neuropathic pain in the trigeminal nerve system, leading to better comprehension and potential treatment options for these pain conditions [82]. This model replicates neuropathic pain by performing a partial ligation (tying-off) of the infraorbital nerve, which is a branch of the trigeminal nerve responsible for sensation in the face. The CCI-ION model mimics facial neuropathic pain, which is characterized by increased sensitivity to both touch and thermal hyperalgesia. Using this model, researchers have found that both TRPV1 channels and purinergic signaling play a role in initiating trigeminal neuropathic pain [83,84]. Studies have further revealed that blocking the glutamate receptors effectively reduced pain caused by CCI-ION, suggesting them as promising targets for therapeutic interventions [85,86]. This new model simplifies the surgery, ensures more consistent nerve damage between animals, and minimizes harm to nearby tissues.

#### 5.3.4. The Chronic Compression of Dorsal Root Ganglion (CCD) Model

The CCD model replicates neuropathic pain by implanting a device that applies continuous pressure on the dorsal root ganglion (DRG). This pressure induces neuronal damage and elicits pain-related behaviors [87,88]. CCD-induced pain is unique due to including mechanical allodynia and sensory disruptions. Neuropathic pain arises from altered function in the DRG. This is caused by significant changes in the proteins that control how nerve signals flow (ion channels) and by the increased activity of these cells [89]. Through investigations utilizing these models, researchers have elucidated changes in neuronal excitability, the expression of ion channels, and neuroinflammatory processes that lead to the initiation and chronicity of neuropathic pain.

These models, often utilizing nerve injury techniques, successfully replicate different components of chronic pain observed in humans, enabling researchers to investigate the complex mechanisms involved. This has resulted in notable improvement in the pain drug discovery process. Nonetheless, a significant limitation arises from the inherent differences in nervous system physiology between animals and humans. Recent advancements have been focused on enhancing existing models by incorporating more developed pain behaviors that better align with the human pain experience. Moreover, researchers are exploring alternative models that utilize genetic modifications to simulate specific pain disorders [90]. While limitations exist due to species differences, the current neuropathic animal models offer valuable insights, and ongoing refinements hold promise for even more precise pain research. These models continue to propel our understanding of this complex condition, paving the way for improved treatments and a better quality of life for those suffering from neuropathic pain.

#### 5.3.5. STZ-Induced Diabetic Neuropathic Pain Model

Diabetic neuropathy is a common complication of diabetes, characterized by chronic pain and sensory deficits. Patients with DNP always exhibit spontaneous and stimulus-evoked pain. Diabetic neuropathic pain is a complex condition with many contributing factors. One factor that may play a role is glycinergic neurotransmission. When this process is impaired, pain sensitivity increases, suggesting its importance in nociception, the perception of pain [91,92]. The streptozotocin (STZ)-induced diabetic neuropathic pain model is widely used in preclinical research to study the pathophysiology of diabetic neuropathy and evaluate potential therapeutic interventions.

Chronic hyperglycemia induced by STZ results in oxidative stress, inflammation, and mitochondrial dysfunction, contributing to neuronal damage. The increased expression of voltage-gated sodium channels (e.g., Nav1.3, Nav1.6, Nav1.9) in dorsal root ganglion (DRG) neurons enhances neuronal excitability and ectopic firing, leading to pain. Elevated levels of pro-inflammatory cytokines (e.g., TNF-α, IL-1β) and the activation of glial cells exacerbate neuropathic pain. A recent study using various techniques found that there are more Ca^2+^-permeable AMPAs (CP-AMPARs) in the spinal dorsal horn of people with diabetic neuropathy. This increase is associated with increased pain sensitivity and suggests that CP-AMPARs are important to how the spinal cord processes pain in this condition [93,94]. Another study was conducted to evaluate streptozotocin-induced diabetes as a neuropathic pain model for allodynia and vulvodynia in female rats. The study found that both systemic and topical gabapentin treatments effectively reduce pain in patients with static and dynamic vulvodynia, further validating the usefulness of this model for studying both allodynia and vulvodynia [95].

A new method using ultra-high field magnetic resonance neurography (MRN) was developed to non-invasively study major peripheral nerve segments in diabetic mice. It was further evaluated to investigate the structure and function of peripheral nerves in a mouse model of diabetes. MRN offers the advantage of direct comparisons between the model and human disease, making it a valuable tool for research into diabetes and other peripheral neuropathic conditions [96]. The research findings from this study contribute to a deeper understanding of the nerve damage that occurs in diabetic peripheral neuropathy. Currently, the STZ model is a reliable tool for studying diabetic neuropathy pain, accurately mimicking its metabolic and neuropathic complications in humans and allowing for comprehensive research on both peripheral and central mechanisms of pain. The STZ-induced neuropathic pain model accurately represents key features of human diabetic neuropathy, making it a valuable tool for studying pain mechanisms. However, it may not fully capture the complexity of the human condition. Despite its limitations, the STZ model remains a cornerstone in pain research, providing a reliable platform for understanding and treating diabetic neuropathy.

#### 5.3.6. Burn Injury-Induced Pain 

Burns frequently cause severe, immediate pain and persistent sensitivity to touch, and can be life-threatening. Managing pain in burn patients is a significant medical challenge. A common laboratory model involves immersing a mouse’s paw in hot water for 3 s to induce burns (65 °C ± 0.5 °C) [97,98]. The burn injury pain model involves inducing a controlled burn injury in animal subjects, typically rodents, to study the resulting pain and its underlying mechanisms. Recent studies highlight the role of epigenetic modifications, such as histone post-translational modifications, in regulating pain perception following burn injuries [99]. This model is used to mimic the clinical scenario of burn-induced pain in humans and to test the efficacy of potential analgesic compounds. Recent research suggests that reducing the activity of Nav1.7, a type of sodium channel in nerve cells, could improve pain control in burn patients. A study found that a Nav1.7 blocker or morphine reduced pain-related changes in the spinal cord after burns. Burns damage nerve endings, leading to pain. The simultaneous activation of certain nerve receptors can contribute to chronic pain. This is often accompanied by central sensitization, a heightened sensitivity to pain caused by changes in the nervous system. A recent study using genetically modified mice found that Nav1.7 plays a key role in heat-related pain after burns, but not in pain caused by touch. These findings suggest that Nav1.7-blocking drugs may be effective in treating pain in burn patients [100]. Another study indicates that Nav1.7, a type of voltage-gated sodium channel, plays a crucial role in the development of hypersensitivity in sensory neurons damaged by burn injuries [101]. This model was further evaluated to investigate the roles of the NGF receptor, tyrosine-receptor kinase A (TrkA), and PKC-epsilon in burn-induced primary mechanical hyperalgesia. This burn injury pain model was further evaluated to investigate the role of the nerve growth factor receptor TrkA and protein kinase C-epsilon in burn-induced hypersensitivity to touch. Our findings suggest that nerve growth factor and protein kinase C contribute to the sensitization of nerve cells after thermal injury. This is significant because there is limited understanding of the mechanisms underlying burn injury pain [102].

The burn injury model offers a comprehensive platform for studying pain mechanisms and developing treatments by simulating human burn pain and allowing for the examination of both its peripheral and central factors. The use of animal models to study burn injuries raises ethical concerns due to the potential for suffering. However, the similarity between animal and human burn pain makes these models valuable for understanding pain mechanisms. By examining molecular and cellular processes in animals, researchers can potentially develop new treatments for burn pain. The burn injury model has already contributed to our understanding of pain and the creation of new pain medications. While there are differences between animals and humans, the model’s ability to replicate key aspects of human burn pain makes it a valuable tool for preclinical research. Studies using this model have identified potential biomarkers and drug targets that may be relevant to human pain management [103].

### 5.4. Cancer Pain Mechanisms Through Animal Models

One of the most frequent distressing and debilitating symptoms encountered by oncology patients with advanced disease is cancer pain. Cancer pain is not a single, well-defined condition. Instead, it is a complex mix of various pain syndromes, each influenced by different biological processes [104]. It results from tumor expansion, invasion, the anatomical site of the malignancy, and the consequences of the treatment. Understanding the mechanisms behind cancer pain using suitable animal models is essential to developing effective pain treatment strategies and alleviating the suffering of cancer patients [105]. To comprehend the distinct neurochemical mechanisms underlying cancer pain, researchers heavily depend on animal models that simulate this intricate condition. This review examines recent research discoveries and evaluates the advantages and drawbacks of these animal models.

#### 5.4.1. Syngeneic Tumor Implantation Model of Pain

The syngeneic tumor implantation model has become an essential tool in the study of cancer, enabling researchers to investigate tumor biology, evaluate treatment options, and create personalized therapeutic strategies. This model replicates cancer growth and invasion by subcutaneously or orthotopically implanting tumor cells from the same strain of the host. As a result, this model causes chronic pain, hyperalgesia, and allodynia [106]. This model provides an environment for investigating the complex interplay between tumor growth and the unfolding of pain. The neurological pathways involved and possible targets for intervention can be revealed by using this model to analyze the molecular and cellular processes that contribute to tumor-induced pain.

The syngeneic model, in contrast to conventional pain models, makes use of immunocompetent animals and closely resembles the scenario experienced by humans [107]. This makes it possible for researchers to examine pain in a setting that more closely mimics the interactions between the neurological system, the immune system, and cancer. Additionally, it enables researchers to delve into how nerve sensitization and pain transmission are impacted by tumor-related variables. By studying pain pathways linked to tumor growth in animal models, researchers can pinpoint potential therapeutic targets. These targets hold promise for developing effective treatments to manage pain in cancer patients [108,109]. Researchers have utilized the syngeneic model to shed light on the crucial role of neural plasticity in cancer pain. This model effectively demonstrates how nerve sensitization alters pain perception. These discoveries not only improve our understanding of how we perceive pain, but they also pave the way for entirely novel pain-relief treatments that will help both cancer patients and people with other types of long-term pain.

#### 5.4.2. Bone Metastasis Model of Pain

The Bone metastasis model is primarily used to research the pain brought on by bone metastases, which commonly develop in advanced stages of cancer. In this model, the introduction of cancer cells into the bone marrow causes bone loss and pain that is related to malignancy. Bone metastasis pain is unique in that it includes spontaneous pain, increased sensitivity to pain (hyperalgesia), and even pain from light touch [110,111]. The significance of brain plasticity in amplifying pain signals has been highlighted in studies using bone metastasis models, offering insight into the mechanisms behind chronic pain. Several investigations have shown that bone remodeling and osteoclast activation contribute to cancer-induced bone pain, pointing to potential targets for pain management. This model has also shed light on how neuroinflammation affects bone pain, opening the door for anti-inflammatory treatments [112]. This research model aided scientists in discovering that neurotrophic molecules (like NGF and BDNF) play an important role in causing bone pain resulting from cancer [113]. According to a recent study, neuropeptides play a role in modulating cancer-induced bone pain [114]. Additionally, research has shown that abnormalities in peptidergic signaling may impair the motor coordinator and have the potential to treat pain brought on by bone metastases.

#### 5.4.3. Development of Colorectal Carcinoma Metastasis Model of Pain

Continually posing a serious threat to global health is colorectal carcinoma, a common cancer of the colon and rectum. A surge of recent research has explored the intricate mechanisms behind colorectal cancer’s spread and the associated pain [115]. To explore cancer-induced pain linked to abdominal organ metastases, researchers employ the colorectal cancer metastatic model. This model involves injecting colorectal cancer cells into the peritoneal cavity, which causes the growth of metastases in visceral organs, resulting in pain. For preclinical pain research, murine models with functional immune systems are crucial. This ensures a closer resemblance to human cancer, allowing for a more reliable translation of findings when identifying new pain treatment targets [116]. Another study developed a colorectal cancer-on-chip, a miniaturized model of human colorectal cancer, complete with adjustable features. This innovative tool holds promise for studying the early stages of cancer spread and the pain associated with it [117]. In addition, investigations have shown that inhibiting inflammatory mediators and targeting TRP channels can reduce pain brought on by colorectal cancer metastasis [118,119]. New therapeutic approaches could develop as scientists continue to improve their understanding of the intricate systems that drive pain in metastatic colorectal cancer, providing comfort and hope for individuals battling this aggressive disease.

### 5.5. Genetically Modified Pain Models

Genetically modified animal models play a crucial role in comprehending pain mechanisms, advancing the development of novel therapeutic interventions, and assessing the effectiveness and safety of pain medications before conducting human trials. Scientists create these models by genetically modifying them to target specific genes or signaling pathways known to influence pain perception and the primary role of pain sensation. Genetically modified animals offer valuable clues for scientists in identifying the genes and molecules involved in sensing, transmitting, and controlling pain [120,121].

#### 5.5.1. TRPV1 Knockout Mice

Transient receptor potential vanilloid 1 (TRPV1) channels are polymodal nociceptors that respond to various noxious stimuli, including heat, capsaicin, and protons. Their activation plays a pivotal role in pain sensation, making them a promising target for pain management. TRPV1 knockout mice, lacking functional TRPV1 channels, have been extensively used to investigate TRPV1’s role in pain pathways and to evaluate the therapeutic potential of TRPV1 antagonists [122]. TRPV1 knockout mice exhibit reduced sensitivity to noxious heat and capsaicin, indicating TRPV1’s essentiality for these pain modalities. Although TRPV1 knockout mice show diminished nociception, the in vivo impact of persistent TRPV1 activation remains to be completely understood. A recent study compared wild-type and TRPV1 knockout mice using acute nociceptive tests and chronic neuropathy models. The study suggested that the absence of TRPV1 may lead to the decreased production of sensory neuropeptides such as somatostatin, contributing to the increased neuropathic hyperalgesia observed in TRPV1 knockout animals. Moreover, the study proposed that both TRPV1 antagonism and agonism could be therapeutically exploited for the treatment of analgesia due to TRPV1’s opposing functions in different nociception models [123].

While knockout studies have provided valuable insights, knockdown approaches have demonstrated TRPV1’s modulation of stimulus modalities beyond the thermal modality. The contrasting results between knockout and knockdown studies suggest functional differences between the gene deletion and gene silencing approaches. TRPV1’s involvement in pain pathways has led to the development of TRPV1 antagonists as potential therapeutic agents for pain management. Several TRPV1 antagonists have been tested in preclinical and clinical studies, with promising results [124,125]. However, TRPV1 knockout mice have limitations. Compensatory changes in other nociceptor channels may affect their pain phenotype, and the relevance of the TRPV1-mediated pain mechanisms in these mice to human pain conditions may vary. Future research should focus on developing more refined TRPV1 knockout models, investigating interactions between TRPV1 and other nociceptor channels, and evaluating the long-term effects and potential adverse effects of TRPV1 antagonists.

#### 5.5.2. OPRM1 Knockout Mice

The regulation of pain involves the significant involvement of opioid receptors, particularly the mu-opioid receptor (OPRM1), which has been extensively studied in research. This receptor is well-known for its established involvement in pain modulation as well as mediating the effects of opioid painkillers. The mu-opioid receptor, encoded by the OPRM1 gene, holds a central role in the modulation of pain [126]. The OPRM1 knockout mice model of pain has been an invaluable tool in advancing our understanding of the mu-receptor’s role in pain regulation. To gain further insights into the OPRM1 receptor’s function, scientists have developed knockout mouse models that target this gene [127]. Studies using OPRM1 knockout mice, where the gene is deleted, have demonstrated the importance of this receptor in mediating analgesia and the progression of opioid tolerance and dependence [128]. Studies have revealed that OPRM1 knockout mice exhibit increased sensitivity to painful stimuli, including thermal, mechanical, and chemical nociception. This heightened sensitivity further highlights the receptor’s involvement in regulating pain responses. The absence of functional OPRM1 receptors appears to disrupt the endogenous opioid system’s ability to modulate pain perception, leading to dysregulation in nociceptive processing [129]. The discoveries derived from the OPRM1 knockout mice model have profound implications for comprehending the role of the mu-opioid receptor in pain pathways. These findings can contribute to the development of more precise and potent analgesics that target the mu-opioid receptor.

#### 5.5.3. TRPV1 Overexpression Mice

The role of the TRPV1 receptor in pain modulation, particularly its involvement in nociceptive signaling, is being investigated by researchers using genetically modified mouse models that express TRPV [130]. These models involve the overexpression of the TRPV1 gene in sensory neurons, resulting in an upregulated abundance of TRPV1 channels in peripheral tissues and the spinal cord. The overexpression of TRPV1 in mice has led to enhanced pain sensitivity and increased sensitivity to thermal pain [131]. The targeting of TRPV1 as a therapeutic approach has demonstrated promising results, and several TRPV1 antagonists have been developed to mitigate pain in preclinical investigations. Researchers have noted that elevated TRPV1 expression plays a role in the progression and persistence of neuropathic pain conditions. These findings highlight the vital role of TRPV1 as a potential way to treat neuropathic pain [132,133]. TRPV1 overexpression mice models have played a crucial function in elucidating the role of TRPV1 in pain sensation and providing valuable insights into pain processing and modulation. However, further research is required to overcome the challenges associated with TRPV1-based therapies and establish safe and effective treatments for pain management.

#### 5.5.4. Nav1.7 Knock-In Mice

Studies using Nav1.7 knock-in mice have demonstrated that gain-of-function mutations in the Nav1.7 channel led to enhanced pain sensitivity. The voltage-gated sodium channel Nav1.7 plays a central role in pain signaling [134]. Nav1.7 gain-of-function mutations have been linked to inherited erythromelalgia, a rare pain disorder. The generation of Nav1.7 knock-in mice expressing human gain-of-function mutations has provided significant advancements in our understanding of pain processing and the identification of potential therapeutic targets [135]. Nav1.7 knock-in mice expressing human Nav1.7 gain-of-function mutations exhibit increased sensitivity to thermal stimuli, mechanical allodynia, and hyperalgesia, mimicking the symptoms observed in individuals with Nav1.7 channelopathies [136]. The utilization of Nav1.7 knock-in mice has provided an increased understanding of the involvement of Nav1.7 channels in diverse populations of sensory and sympathetic neurons, which contributes to distinct pain sensations. Mice engineered to lack Nav1.7 only in their pain-sensing neurons (nociceptors) become completely insensitive to both extreme heat and harsh touch. Interestingly, when Nav1.7 is missing only in their sympathetic neurons, these mice still feel mechanical pain but lose sensitivity to heat pain [137]. These studies demonstrate the value of using Nav1.7 knock-in mice to research how this channel affects persistent pain conditions like inflammatory and neuropathic pain. Researchers have learned more about Nav1.7’s function in chronic pain conditions by selectively manipulating it in these animals. The use of Nav1.7 knock-in mice has significantly contributed to the progress of our knowledge regarding pain processing and the involvement of Nav1.7 sodium channels in nociception. These studies have provided valuable insights into the molecular foundations of pain, opening possibilities for the development of novel and targeted pain treatments in the future.

#### 5.5.5. P2X3 Knockout Mice

The P2X3 receptor is an ion channel activated by ATP and is primarily found in sensory neurons. The use of P2X3 knockout mice has been extensively employed in pain research to investigate the functions of the P2X3 receptor in pain perception and processing. P2X3 knockout mice have been employed as a research model to examine the role of P2X3 in persistent pain conditions, including pain from visceral and neuropathic regions. These mice exhibit diminished responses to a wide range of noxious stimuli, such as mechanical, thermal, and chemical pain [138]. This decreased sensitivity suggests that the P2X3 receptor is essential in transmitting nociceptive signals and modulating pain perception. P2X3 knockout mice display altered sensory processing in response to a variety of pain stimuli. The lack of P2X3 receptors impacts the transmission of nociceptive information from the peripheral regions to the central nervous system, leading to a decrease in nociceptive signaling [139]. This finding further emphasizes the significance of the P2X3 receptor in sensory processing and nociception [140]. A group of researchers investigated a new antagonist called A-317491, which effectively blocked the activity of P2X3 and P2X2/3 receptors by inhibiting calcium flux. The antagonist demonstrated a distinguished specificity for these receptors and successfully alleviated pains from thermal hyperalgesia and mechanical models of nerve injury and chronic inflammation. Overall, the study highlights the potential therapeutic use of targeting P2X3 and P2X2/3 receptors for managing chronic pain conditions [141]. P2X2/P2X3Dbl −/− mice lack both P2X2 and P2X3 receptors, resulting in no taste preferences or aversion to bitter substances like sodium chloride or SC45647, unlike wild-type mice. This suggests that their taste nerves do not respond to these substances. These mice have helped establish the significance of P2X3 receptors in nociceptive signaling, sensory processing, inflammatory pain, and visceral pain.

Developing a thorough grasp of the involvement of P2X3 receptors in pain modulation could have significant implications for the advancement of innovative therapeutic approaches to managing pain. Additionally, the P2X2 and P2X3 purinergic receptors play a crucial role in transmitting taste information from the taste buds to the gustatory nerves. The molecular and cellular mechanisms underlying the processing of pain have been clarified in part thanks to these genetically altered animal models of pain. Researchers have learned a lot about the physiology of pain via the study of these models, and this knowledge has implications for the creation of future pain medicines that are more effective and precise. The ongoing use of these genetically altered pain models in the study of the processing of pain holds promise for the creation of more efficient and individualized pain treatments, ultimately enhancing the overall well-being of millions of people with chronic pain disorders.

#### 5.5.6. Application of CRISPR in Pain Research: A Promising Frontier

Despite extensive research, the underlying mechanisms of and effective treatment options for chronic pain remain limited. The emergence of CRISPR-Cas9 technology has revolutionized the field of molecular biology and holds great promise for advancing pain research. Researchers can utilize CRISPR technology to gain a valuable understanding of the molecular foundations of pain, which can subsequently facilitate the development of more precise and effective approaches for pain management. By selectively disabling specific genes using CRISPR, researchers have been able to determine the impact of gene knockout on pain responses in animal models. This approach helps to confirm the relevance of specific genes as therapeutic targets and enables the development of more targeted and effective pain medications. The advent of CRISPR-Cas9 technology provides an unprecedented opportunity to unravel the complex genetic and molecular bases of pain. This section aims to highlight recent advancements in CRISPR-based approaches and their potential impacts on pain research.

#### 5.5.7. CRISPR on Identification of Novel Pain Targets and Elucidating Pain Pathways

CRISPR-based high-throughput screening methods have proven invaluable in identifying novel genes and molecular pathways involved in pain perception. For example, a recent study utilizing CRISPR technology identified a previously undiscovered gene linked to neuropathic pain. This groundbreaking discovery offers valuable insights into the underlying molecular mechanisms of this condition [142]. CRISPR-mediated gene editing enables the precise manipulation of specific genes or regulatory elements, allowing researchers to investigate their roles in pain pathways. By selectively modifying genes involved in pain transmission, modulation, or sensitization, researchers can dissect the complex interplay between various components of pain signaling cascades. Another recent study demonstrated the importance of a specific ion channel in the regulation of chronic pain, shedding light on potential therapeutic targets [143]. By targeting genes involved in pain sensitization, inflammation, or neural plasticity, CRISPR-based therapies have the potential to mitigate chronic pain. Numerous experiments have explored the therapeutic potential of CRISPR for use in preclinical models, demonstrating successful pain relief by targeting key pain-related genes [144,145]. To successfully implement CRISPR-based pain therapies in clinical settings, additional research and the improvement of delivery systems are necessary to guarantee both safety and effectiveness. Moreover, CRISPR-based screening approaches have been employed to identify novel targets for pain drug discovery. Genome-wide CRISPR screens have facilitated the identification of genes that modulate pain-related phenotypes, offering potential druggable targets for pain relief. Through these investigations, valuable insights have been gained into the intricate molecular mechanisms that underlie pain. This knowledge serves as a roadmap for the establishment of personalized pain therapies which can precisely target specific pain pathways [146,147].

CRISPR-Cas9-mediated genome editing was used to identify the α2δ-1 subunit of voltage-gated calcium channels as a therapeutic target for pain [148]. This study used CRISPR-Cas9 to investigate the role of the α2δ-1 subunit in pain transmission. By targeting and editing this gene in mice, the researchers displayed a substantial decrease in pain sensitivity, suggesting that α2δ-1 could be a potential target for pain drug development. Researchers employed CRISPR interference (CRISPRi) to study the gene regulation that takes place when persistent pain is experienced. By suppressing specific genes associated with pain signaling, they were able to alleviate pain-related behaviors in mice. This finding highlights the potential of CRISPRi as a tool for discovering new targets for pain mediation [149]. Another study applied CRISPR-based epigenome editing to enhance neuronal differentiation and axon growth. By modifying the epigenetic regulation of cytokine receptors, the researchers were able to promote neuronal development, which has implications for nerve regeneration and pain management [150].

These scientific findings showcase the potential of CRISPR technology in advancing our understanding of pain pathways and identifying new targets for pain drug discovery. CRISPR has accelerated the identification of pain-associated genes and potential drug targets. Moreover, the development of patient-specific models using CRISPR-mediated gene editing has opened up new avenues for personalized pain medicine. The continued investigation of CRISPR in pain research shows potential for the advancement of pain therapeutics that are both safer and more effective. It is important to note that these findings represent a subset of the extensive research conducted in CRISPR and pain drug discovery. In conclusion, recent research findings demonstrate the transformative potential of CRISPR technology in pain drug discovery.

### 5.6. Cellular Models of Pain

**Figure 4:**
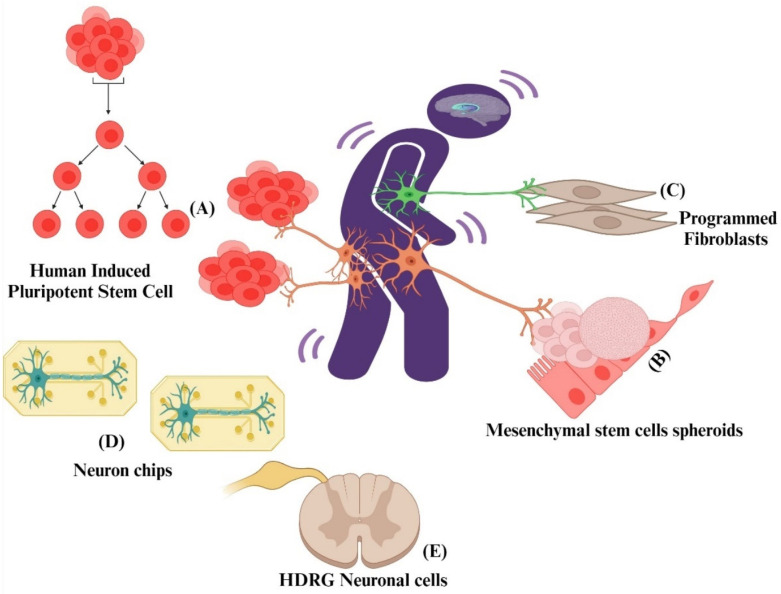
The major types of cellular models that are used to evaluate pain. (**A**) Human-induced pluripotent stem cells (hiPSCs)—These have enabled the generation of various difficult-to-access cell types such as human nociceptors; (**B**) MSC-Spheroids have been shown to alleviate neuropathic pain by modulating the activity of genes associated with chronic inflammation. Mesenchymal stem cells (MSC)—spheroids are popular cellular tools in the field of pain research. (**C**) Programmed Fibroblasts—researchers are using nociceptor neurons derived from fibroblasts to investigate pain mechanisms in a controlled laboratory setting. (**D**) Neuron chips—researchers have developed nerve-on-a-chip models to study neuronal functions in a simplified and controlled environment; (**E**) DRG-N- human dorsal root ganglia neuronal cells—these neurons are the gold standard biological agent for researchers investigating pain and sensory processing. These cells have been used in the treatment of nociceptive pain, neuropathic pain, and nociplastic pain, and their effectiveness and safety have been validated in numerous preclinical studies and clinical trials [153]. All images in this figure were created with BioRender.com, BioRender, Canada (BioRender.com).

Animal models have demonstrated significant aspects of pain neurobiology, but the clinical integration of these data necessitates our understanding of human cells and the neurological basis of pain in humans [151]. Although cell-based studies in pain research are still in the early stages, further explorations into the mechanisms of neuronal interactions within pain pathways can potentially pave the way for the development of novel medications to address both acute and chronic pain conditions. The biggest hurdle in developing novel drugs for pain and treating it is the elucidation of mechanisms involved in neuronal cell-injury-based pain [152]. Exploring the potential association between neuronal cells and pain through in vitro cell-based pain assays, such as those involving Schwann cells, can offer valuable insights into the mechanisms involved in amplifying pain signals. These investigations may also provide valuable clues to guide the development of innovative therapeutic agents for effective pain management. Figure 4 illustrates an overview of different cell-based models that researchers use to study pain and develop novel pain compounds.

Schwann cells (SCs) and their axon connections play multiple key roles in nerve formation, stabilize the axonal transport mechanism, and provide trophic support; additionally, new evidence shows that SCs play a critical part in controlling neuropathic pain, which is believed to be a major consequence of nerve injury [154,155]. It has been demonstrated experimentally in transgenic mice that the disruption of fibroblast growth factor receptor (FGFr) signaling in SCs has been implicated with sensory axonal neuropathy and the impairment and modulation of pain sensory pathways. This study on the SCs of transgenic mice revealed a putative molecular mechanism for pain, with possible implications for nociception in numerous neuropathies [156]. An in vivo investigation concluded that paw tissue and cultured mice SCs express TRPA1 to cause oxidative stress, which targets neuronal TRPA1 to maintain allodynia. This is corroborated by acetaldehyde (ACD) levels that were dose-dependent in cultured Schwann cells obtained from C57BL/6J mice and treated with ethanol. These findings have been confirmed by the observation of TRPA1 mRNA expression and sustained elevations in intracellular calcium in human Schwann cells (HSCs) [45]. In a murine CCI model, conditional ablation of the MHC-II β subunit in myelinating Schwann cells (SCs) resulted in the attenuation of both thermal hyperalgesia and mechanical allodynia. This finding corroborates prior experimental evidence implicating SCs in peripheral nerve injury and the development of neuropathic pain [157].

Pannexin 1 (Panx 1) is an essential membrane channel that regulates neuroinflammation and contributes to the secretion of ATP and inflammatory cytokines in primary SCs during neuropathic pain. In cultured primary mouse SCs, increased cytokines (IL-1, IL-6, and TNF-α) and mRNA were found, which elucidated the role of Panx 1 in the inflammatory response and neuropathic pain via the production of selective cytokines [158]. Another transcription factor, Sox10, is primarily expressed in HSCs and has been linked to SC homeostasis, pain initiation in skin, pain hyperalgesia, and peripheral neuropathy [159,160,161]. The growth factor neuregulin 1 type III supports and expresses HSC survival and differentiation via its receptors (ErbB2 and ErbB3). Recent findings indicate that the loss of these nociceptive SCs is sufficient to elicit neuropathic-like pain in the mouse model, necessitating more research into the roles of Sox 1 and neuregulin 1 [151,162]. To fully elucidate the critical role of nociceptive hematopoietic stem cells (HSCs) in both axon integrity and pain sensitization, further comprehensive investigations are warranted. These studies should utilize well-validated animal models of neuropathic pain or established cell-based assays specifically designed to mimic this condition. A greater understanding of these HSC-based pain assays and neural communications may help to uncover new mechanisms of pain signaling for drug discovery, advancing pain therapy for people worldwide. This finding underscores the need for further investigation into the mechanisms by which nociceptive Schwann cells (SCs) in human skin contribute to pain perception. Additionally, exploring their potential function in the emergence of neuropathy and hyperalgesia using validated animal models could provide valuable insights.

#### 5.6.1. Reprogrammed Nociceptor Neurons from Fibroblasts

In recent years, there has been growing interest in developing in vitro models to study pain mechanisms using nociceptor neurons that have been reprogrammed from fibroblasts. Several studies have successfully reprogrammed fibroblasts into functionally mature nociceptor-like neurons using various reprogramming techniques, including direct lineage conversion and induced pluripotent stem cell (iPSC) technology [163]. These reprogrammed nociceptor neurons exhibit characteristic nociceptive properties, such as responsiveness to noxious stimuli and the expression of pain pathways including ion channels and receptors. The utility of patient-derived neurons as a tool for drug screening would be maximized if these neurons were to accurately replicate the sequence of pathophysiological events leading to specific pain-associated diseases. The potential exists to generate diverse classes of neurons in vitro by overexpressing specific transcription factors in fibroblasts. This approach holds promise for conducting mechanistic studies of diseases and facilitating drug screening processes [164]. In recent studies, scientists have identified five transcription factors that can convert both mouse and human fibroblasts into neurons that detect noxious stimuli, known as nociceptor neurons [165]. Overall, the utilization of in vitro models with nociceptor neurons reprogrammed from fibroblasts provides a valuable tool for studying pain at the cellular level and offers a promising avenue for future advancements in pain research and therapeutic development. Collectively, these studies demonstrate methodologies for producing functional peripheral sensory neurons from both mouse and human fibroblasts in vitro. Furthermore, they underscore the potential utilization of these cells for modeling pathological conditions.

#### 5.6.2. Human-Induced Pluripotent Stem Cells (HiPSCs)

Human-induced pluripotent stem cells (HiPSCs) have revolutionized biomedical research by providing a versatile platform for disease modeling, drug discovery, and regenerative medicine. HiPSCs are generated by reprogramming somatic cells, such as skin or blood cells, into a pluripotent state using defined transcription factors (e.g., OCT4, SOX2, KLF4, and c-MYC) [166]. These cells can then be differentiated into various cell types, including neurons and glial cells, which are critical for studying pain mechanisms. The HiPSC-derived nociceptors were characterized by the expression of TrpV1, Na1.7, and P2X3, key molecular markers for nociceptive function and pathological pain. Their functional responses to relevant stimuli were validated in a high-content screening system [167]. The process involves the reprogramming and differentiation of somatic cells to a pluripotent state and subsequently differentiating them into specific cell types relevant to pain research. HiPSCs can be differentiated into sensory neurons that express pain-related receptors and ion channels, such as TRPV1 and Nav1.7, allowing for the study of pain signaling pathways [168].

HiPSCs can be derived from patients with specific pain conditions, enabling the study of genetic and molecular mechanisms underlying individual pain phenotypes. HiPSCs bypass ethical issues associated with embryonic stem cells, as they are derived from adult tissues. They can be differentiated into various cell types, providing a comprehensive platform for studying different aspects of pain [169]. However, the efficiency of reprogramming somatic cells into HiPSCs can be variable, which may affect the consistency of the models. Differentiated cells may not fully recapitulate the maturity and functionality of native cells, potentially limiting their utility in modeling complex pain mechanisms. Despite this, HiPSC-derived sensory neurons, exhibiting key features of human pain pathways, offer a face-valid model for studying pain mechanisms, demonstrating predictive validity for drug discovery, and providing cellular and molecular insights into potential therapeutic targets. HiPSCs offer a unique opportunity to model human pain conditions in vitro. By generating patient-specific HiPSCs, researchers can study the genetic and molecular bases of pain, identify biomarkers, and screen for potential analgesic drugs. This approach has the potential to bridge the gap between preclinical studies and clinical applications, leading to more effective and personalized pain treatments [170]. Their ability to model human pain pathways, combined with their ethical advantages and versatility, makes HiPSCs an invaluable resource in the quest to understand and alleviate pain. Despite certain limitations, the strengths and validity of HiPSC models underscore their potential to transform pain research and lead to the development of novel analgesics.

#### 5.6.3. Human and Rat hDRG Neuronal Cultures for Pain Drug Discovery

Human and mouse cell models have the potential to be used to validate novel pain targets and develop promising analgesic molecules along the translational pathway before investing precious research resources towards clinical development. The concomitant necessity for cellular models has increased the demand for the rapid acceleration of mouse and human sensory neuron cultures in pain drug development in recent decades. In preclinical pain research, in vitro neuronal cell culture models bridge the gap between complex in vivo animal models and human patients. These models offer a controlled environment in which to meticulously study the molecular pathways underlying pain [171]. Primary cultures of DRG are commonly used to research the cellular underpinnings of nociception, and they circumvent many of the constraints correlated with in vivo pain models. DRG primary cultures are a significant experimental tool used to investigate the cellular bases of pain-related studies, and they circumvent many of the constraints associated with in vivo animal pain models [172].

Experimental evidence suggests that DRGs from rats and humans exhibit identical voltage-gated Na+ channels (VGSCs) during neuropathic pain and injury, and these are potentially critical for uncovering new pain treatments as well as in understanding the molecular pathways of pain. The recent neuro-physiological and transcriptome investigations of mouse and human DRGs indicate that they have significant implications in pain research and could contribute to the establishment of an in vitro neuropathic pain model. This validates the use of cultured DRG neurons as a model system for researching the fundamental mechanisms of pain and related drug targets. It was also demonstrated that rat DRG neuronal cultures were successfully employed to explore the role of a selective alpha2-adrenoceptor agonist, dexmedetomidine, in the induction of DRG neurons. Apoptosis may be an important way for reactive oxygen species to contribute to neuropathic pain [173,174,175].

By developing models that closely mimic real-world pain experiences, researchers can gain deeper insights into the underlying pathophysiology of these experiences, paving the way for novel pain treatments. Organoids are 3D cell cultures derived from stem cells that can develop into structures resembling organs or tissues, replicating their in vivo morphologies and functions [176]. The emergence of human brain organoids opens exciting possibilities for exploring the mechanisms of pain. By mimicking the intricate workings of the human brain, these organoids hold immense promise for providing novel insights into pain pathogenesis [177]. Sensory neuron organoids have emerged as a significant advancement in pain research. These complex and biologically relevant models provide researchers with the opportunity to explore the intricate mechanisms underlying pain perception and processing in greater depth [178]. The ability to subject these sensory neuron organoids to different stimuli, such as thermal, mechanical, or chemical triggers, enables researchers to study the neurons’ reactions and get a deeper understanding of the molecular signaling involved in pain perception. It is vital to remember that sensory neuron organoids have their limitations, much like any model system. Compared to animal models, they provide a setting that is more relevant to humans, but they still do not capture the complete complexity of a real neurological system. To ensure the therapeutic applicability of their findings, researchers must exercise caution and validate their findings in real patients.

### 5.7. Alternative to Animal Models of Pain

#### 5.7.1. Zebrafish (*Danio rerio*)—A Fish Model

**Figure 5:**
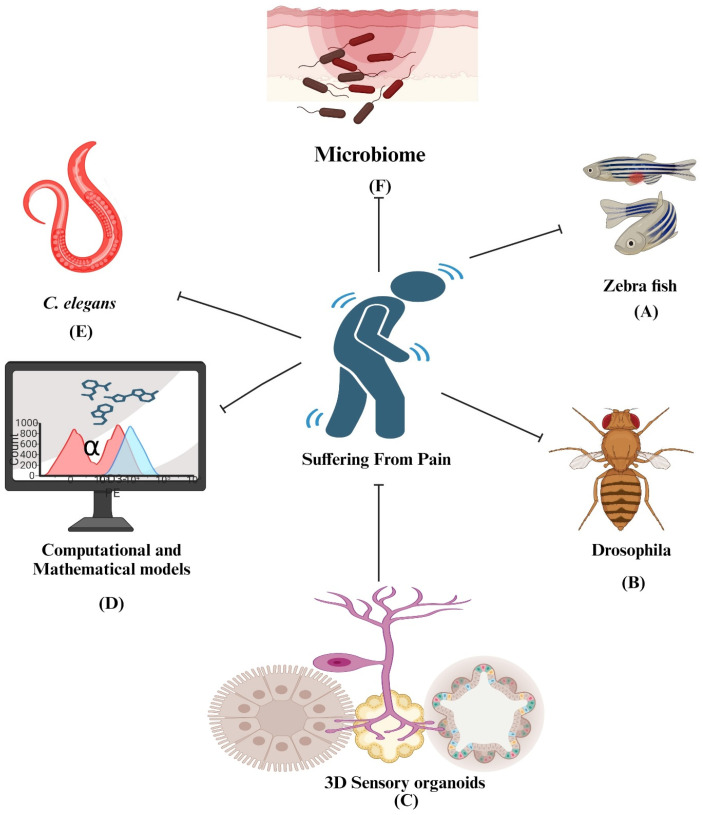
Alternative to animal and human models of pain (created with BioRender.com). (**A**) The zebrafish (*Danio rerio*) presents a complex physiological system that recognizes and responds to painful stimuli [181]; (**B**) *Drosophila melanogaster*—these small, genetically tractable insects have been used as models for studying various biological processes, including pain perception. (**C**) 3D sensory organoids—these 3D organoids are being used to develop new pain treatments. They are human spinal organoid-on-a-chip devices that mimic the biology and electrical activity of human nociceptive neurons and dorsal horn interneurons in pain pathways; (**D**) Computational and mathematical model—computational modeling has emerged as a promising methodology in unravelling the intricate neural mechanisms contributing to neuropathic pain. For peripheral pain specifically, some models focus on the cellular and molecular aspects of how the body processes harmful stimuli, while others use artificial neural networks based on the idea that only these parallel-distributed processors can replicate the computational abilities of the nervous system; (**E**) Caenorhabditis elegans—this is a valuable model for studying pain sensation, as it displays a clear and repeatable withdrawal response, turning away from painful stimuli. (**F**) The gut microbiome—the gut microbiome is a key regulator of visceral pain while emerging evidence suggests its vital involvement in multiple other types of chronic pain, including inflammatory pain, headaches, neuropathic pain, and opioid tolerance [153]. All images in this figure were created with BioRender.com, BioRender, Canada (BioRender.com).

Novel biobehavioral assays using the zebrafish model, which is similar to humans and represents an ideal alternative organism, can discover genetic pathways of pain mechanisms, fundamental aspects of pain pathology, and molecular targets [179]. Recent translational scientific discoveries from the zebrafish (*Danio rerio*) model are becoming increasingly important alternative tools in medical research and have aided in the development of methods to evaluate effective analgesics. The use of alternative mammalian animal models has demonstrated its efficacy and may enable neurobiologists to discover new compounds with potential analgesic activity (Figure 5). A new study on zebrafish revealed a novel molecular perspective on pain perception in response to noxious stimuli which could be used to screen for chemicals with potential analgesic activity. The study also demonstrated that zebrafish larvae can be used to explore cellular and genetic networks associated with pain symptoms in mammals [180].

Another systematic review suggested that pain screening tools in zebrafish be used to test the effectiveness of noxious stimuli and to develop new systematic methods to support potential pain-related preclinical research [182]. A novel technique was devised by scientists to examine pain in zebrafish by employing cryoinjury. While this method demonstrated the effectiveness of morphine in relieving pain symptoms, it was observed that the zebrafish swam at a slower pace following the procedure. This indicates that the cryoinjury itself might have been inducing discomfort or pain, which could potentially impact the precision of pain measurement. Therefore, it may be crucial to further explore alternative approaches for inducing pain in zebrafish to ensure more dependable pain research.

#### 5.7.2. *Drosophila melanogaster*: A Fruit Fly Model

The common fruit fly, *Drosophila melanogaster*, is a powerful tool for scientists studying the senses and pain perception. This tiny fly, belonging to the *Drosophilidae* family, shares a surprising similarity with humans: about 75% of the disease-related genes in humans have counterparts in fruit flies. This remarkable genetic overlap makes the fruit fly an invaluable model organism for unraveling the mysteries of pain [183]. In contrast to mammalian models, *Drosophila* presents unique advantages for pain research, characterized by its small size, short lifecycle, and facile large-scale production. Fruit flies (*Drosophila*) have a surprising number of genes (13) dedicated to a specific family of proteins called TRP channels. Four of these genes even resemble human TRPA channels. These genes are particularly interesting to scientists because they seem to play a key role in how fruit flies sense temperature [184,185]. Numerous studies highlight the intricacy of *Drosophila*’s behavior, suggesting that *Drosophila* flies exhibit sufficient complexity, which qualifies them for utilization in pain drug discovery and related research. Several orphan steroid receptors and two annexins, implicated in cell differentiation and insect development, have been identified in *Drosophila* [186]. *Drosophila melanogaster* larval nociception has been well-characterized using both localized and whole-body mechanical and thermal stimulation [184,187,188]. Hence, it seems that, at present, utilizing a *Drosophila* (fruit fly) model for screening potential analgesics and characterizing antinociceptive mechanisms and novel drug targets would be advantageous [189,190].

In a recent study, a refined *Drosophila* model of mechanical nociception was developed to explore ion channels and signaling pathways governing this process. The study uncovered a preserved signaling pathway involving VEGF-related receptor tyrosine kinases that regulates mechanical nociception in flies [191]. These findings underscore the efficacy of using *Drosophila*’s genetics to swiftly identify and screen novel therapeutic targets with potential clinical significance. Significantly, the research provided evidence for the presence of a conserved receptor tyrosine kinase (RTK) signaling pathway that regulates baseline mechanical nociception in both flies and rats. The study further validated the use of *D. melanogaster* larvae as a model for identifying potential analgesic compounds in response to nociceptive stimuli and pain-induced sensitization. Various analgesic drugs were administered in a 100 nL volume before measuring the nociception during the application of an infrared noxious stimulus. This study found that analgesics specifically target pain sensations (sensory/nociceptive processes) in *D. melanogaster* larvae, without affecting their movement or coordination. This suggests that the *D. melanogaster* larvae model is valuable for discovering new pain medications [192].

A new Ca^2+^ signaling gene (α2δ3/*stj*) was discovered in *Drosophila*, through genome screening for nociception behavior, which functions as a peripheral component of multiple Ca^2+^ channels. The study involved testing α2δ3 knockout mice and revealed impaired responses to noxious temperatures and delayed inflammatory pain sensitization. *Drosophila*, serving as a model organism, has been used to investigate not only heat pain but also mechanical nociception, as observed in the initial *Drosophila* pain study. Additionally, a mutant gene named ‘painless’ has been identified in *Drosophila*, demonstrating antinociceptive responses consistent with pain experiences in both *Drosophila* and *Drosophila* larvae [193].

#### 5.7.3. *Caenorhabditis elegans*: A Nematode Model

*Caenorhabditis elegans* provides a valuable opportunity to study the intricacies of how organisms respond to harmful stimuli. This is due to their well-defined and consistent withdrawal reflexes upon exposure to such stimuli in their environment. While these responses are crucial for understanding nociception, it is important to exercise caution when directly equating them to human pain perception due to the significant differences between nematode and human nervous systems and cognitive abilities. A laser-based assay was recently devised by researchers to measure the heat avoidance response in *C. elegans*. This response, characterized by a distinct withdrawal reflex, was significantly weaker in mutants with altered glutamate signaling, altered neuropeptide function, or an altered sensory neuron structure. These findings demonstrate the assay’s reproducibility and highlight its value for investigating the genetic and pharmacological bases of heat-induced pain responses in *C. elegans*. Importantly, the nematode’s nociceptive responses share significant similarities with those of higher organisms. *C. elegans* has powerful genetic features that could help scientists discover new genes and processes involved in how we feel pain [194,195]. Recent research has employed optogenetics to activate *C. elegans*’ nociceptors, specifically targeting channelrhodopsin 2, found within the PVD nociceptive neurons, to study its role in nociception [196,197,198]. In a recent investigation using *C. elegans*, a novel neuroendocrine pathway was identified that connects pain-sensing TRPV1 channels. This discovery holds potential implications for age-related chronic pain disorders. However, the specific pathways and molecular mechanisms underlying this association with CREB-regulated transcription co-activator 1 (CRTC1) and pain in TRPV mutant worms remain unclear [199].

The study employed a quantitative model to gauge the perceived intensity of heat stimuli by observing the predictable escape behavior of individual *C. elegans* nematodes subjected to an infrared laser. This model allows researchers to quantify the pain-relieving (analgesic) effects of chemical treatments or genetic changes in *C. elegans*. Beyond its application to various model systems, this study aimed to solidify *C. elegans* as a powerful tool for nociceptive research [200]. A computer-assisted method was utilized to analyze the heat avoidance behaviour in 109 *C. elegans* mutants with genes similar to those involved in human pain perception. This allowed the pain researchers to identify mutations that affected the worms’ ability to avoid heat. This study uncovers the intricate network of genes involved in pain perception that is shared between *C. elegans* and humans. *C. elegans* presents a fascinating model for studying how glycoprotein fucosylation influences pain perception, as its glycosaminoglycans closely resemble those of humans.

Since pain signals share similar pathways across species, future studies using human pain genes in *C. elegans* worms could offer valuable insights and pave the way for innovative pain management strategies. The roundworm, *C. elegans*, with its simple genetics and suitability for large-scale behavioral studies, offers a valuable model for deciphering poorly understood human pain genes [201]. Another study, on wild-type and mutant *C. elegans*, discovered that capsaicin, the compound responsible for chilli pepper heat, has time- and dose-dependent pain-reducing effects in the worm. This effect seems to occur through the desensitization of the TRPV channel OCR-2, which is involved in heat-induced pain responses. This landmark study identifies OCR-2 as the target of capsaicin in *C. elegans* and provides the first clear demonstration of its pain-relieving properties in this model organism [202]. In conclusion, the *C. elegans* model shows remarkable potential for pain drug discovery, facilitating the way for exciting research avenues and potentially uncovering novel therapeutic targets for pain management.

### 5.8. Human Experimental Models of Pain

**Figure 6:**
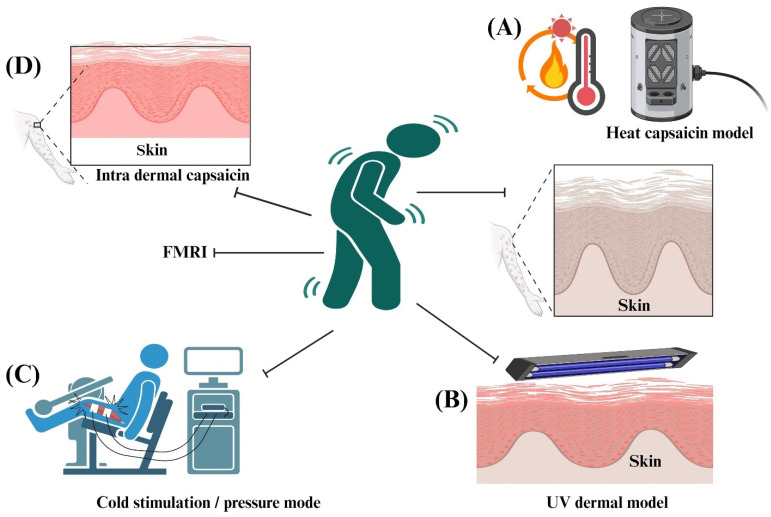
Depicts different human experiment models of pain. (**A**) The heat/capsaicin sensitization model is used to induce heightened sensitivity to pain in the skin. A combination of heat and topical capsaicin is applied to the skin. This model provides a valuable tool for studying the mechanisms underlying pain sensitization and for testing potential treatments for chronic pain conditions; (**B**) The UV-B dermal pain model—by exposing a small area to UV-B radiation, the UV-B pain model creates a controlled inflammatory site. This model is used to evaluate how analgesics affect pain perception, specifically in response to touch and heat; (**C**) Cold Stimulation and Pressor (CSP) model of pain—the cold pressor test is a widely used technique to evaluate pain perception. It involves immersing a body part in cold water, which produces a gradually intensifying pain. Participants can terminate the test by removing the limb. This test is a reliable and safe method for assessing pain in patients before surgery and is commonly used in pain research; (**D**) The intradermal capsaicin model is a widely used tool to assess the efficacy of analgesics. Unlike the heat/capsaicin sensitization model, it produces a brief painful stimulus that is followed by a lasting area of heightened pain sensitivity. All images in this figure were created with BioRender.com, BioRender, Canada (BioRender.com).

The incorporation of human experimental pain can have a significant impact on the advancement of novel analgesic medications. Humanized pain models refer to experimental models or methods used in pain research that attempt to capture the complexity and nuances of pain experienced by humans. These models often involve the use of animals or advanced in vitro systems that mimic human physiological and pathological conditions (Figure 6). The aim of these systems is to better understand pain mechanisms, develop new analgesic drugs, and, ultimately, improve pain management in humans. By modeling pain in humans, researchers gain valuable insights that bridge the gap between preclinical models and the understanding of pain in a clinical context [203]. Although valuable insights have been gained through the extensive use of animal models, the development of reliable pain treatments for humans requires further refinement of these models or the exploration of alternative approaches. Several clinical manifestations of pain cannot be accurately simulated by animal models. Several animal studies have shown a lack of translational effectiveness in understanding pain or delivering new therapies for it. Because of these shortcomings, preclinical research on novel analgesics frequently benefits from the use of human experimental models of pain [204]. Essential for understanding pain mechanisms, human experimental pain models are particularly well-suited for evaluating the effects of compounds targeting pain. The ability of human volunteers to report on their pain experience can be used for pain research. A recent review emphasized the potential of human-based pain research to enhance our understanding of pain conditions [205].

Establishing a collaborative human pain research network could significantly advance volunteer studies, reducing the reliance on animals in pain research. Neuroimaging techniques have contributed valuable insights into the complex brain processes involved in experimental pain. Human experimental pain models may serve as a translational bridge between animal and clinical research. Even these human models of pain, however, only capture a portion of the disease. The significance of these models in pain research has grown steadily, as they enable a deeper comprehension of pain mechanisms, facilitate the development of innovative pain therapies, and provide a means to evaluate potential analgesic medications. By incorporating human elements into animal models, researchers can better translate their findings to the clinic and improve patient outcomes. One promising method to humanize pain models is the introduction of human pain-related genes or cells into animals. For example, researchers have been able to generate transgenic mice expressing human pain-related genes such as Nav1.7, a voltage-gated sodium channel implicated in pain perception [137,206]. By studying these mice, researchers can gain insights into how specific genetic factors influence pain sensitivity and responses. To design more realistic models of human pain, researchers are now including the mental and emotional sides of pain in their animal studies. This involves designing new behaviors that mimic how people think and feel when they are in pain. Thus, the heat/capsaicin sensitization model offers a promising platform for investigating pain mechanisms and evaluating novel analgesic candidates.

#### 5.8.1. The Heat/Capsaicin Sensitization Model

The heat/capsaicin model is known for its reproducibility and stability. The heat/capsaicin sensitization model uses a combination of heat and topically applied capsaicin to cause cutaneous sensitization, which works through the transient receptor potential vanilloid 1 receptor [17]. The principal mechanism of the heat/capsaicin sensitization model involves the application of heat followed by capsaicin to the skin. The heat sensitizes the skin, making it more responsive to capsaicin, which activates the transient receptor potential vanilloid 1 (TRPV1) channels on the nociceptive neurons. It can generate consistent and long-lasting areas of hyperalgesia and secondary hypersensitivity, making it a reliable tool for studying pain mechanisms and testing analgesic drugs. Capsaicin binds to the TRPV1 channels, which are non-selective cation channels located on the membranes of nociceptive neurons, and this binding causes the channels to open, allowing calcium and sodium ions to flow into the cell. The initial application of heat increases the sensitivity of the TRPV1 channels to capsaicin. This sensitization is due to the phosphorylation of the TRPV1 channels by protein kinase C (PKC) and other kinases, which lowers the activation threshold of these channels. As a result, the neurons become more responsive to subsequent capsaicin application, leading to enhanced pain perception [207,208]. To induce cutaneous sensitization (increased skin sensitivity), researchers utilize the heat/capsaicin model, which combines heat exposure with the topical application of capsaicin. The sensitization induced by this model can last for several hours, providing a sufficient window to test the efficacy and onset of analgesic interventions.

Researchers designed the heat/capsaicin sensitization model to be a safe, noninvasive way to induce long-lasting primary and secondary hyperalgesia in the skin. This reliable model allows for testing the effectiveness of oral analgesic compounds [209]. Research suggests a synergistic interaction between heat and capsaicin, leading to prolonged sensitization of the skin. Researchers have developed a new pain model, CHOP (capsaicin/heat ongoing pain), that mimics key features of chronic pain like a longer duration, sensitization, and heightened sensitivity to touch (allodynia) and pain (hyperalgesia). Unlike existing models, CHOP can be safely applied for up to an hour without causing tissue damage. This innovative approach allows scientists to investigate various pain mechanisms, including both initial pain (primary hyperalgesia) in the area of injury and pain that worsens over time in surrounding undamaged tissues (secondary hyperalgesia). Additionally, CHOP enables the study of how psychological factors influence pain perception. These features make CHOP a valuable tool for addressing real-world pain questions in future research [210]. However, a methodological study by Dirks and colleagues concluded that there was no synergistic or additive effect between heat and capsaicin in inducing cutaneous sensitization [211]. An interesting finding from the study is that rekindling the stimulus plays a leading part in maintaining prolonged skin sensitization [212].

To evaluate the reproducibility of the findings, the researchers conducted additional assessments by manipulating the size, duration, and intensity of the noxious stimulation. These factors play a crucial role in inducing and sustaining areas of secondary hyperalgesia in various conditions [213]. By applying the essential “ongoing nociceptive input” to a smaller surface area than previously reported, this study provided the initial evidence revealing a failure to replicate the original heat/capsaicin cutaneous sensitization model. The model closely mimics clinical conditions of inflammatory pain, making it highly relevant for translational research. It helps bridge the gap between preclinical studies and clinical trials. However, studies have shown that there is no synergistic or additive effect between heat and capsaicin in inducing cutaneous sensitization. This limits the model’s ability to fully replicate the complexity of clinical pain conditions. In conclusion, the heat/capsaicin sensitization model is a valuable tool in pain research, offering several advantages in terms of reproducibility, stability, and clinical relevance. However, its limitations, including the lack of synergistic effects and gender variability in responses, must be considered when interpreting results and designing experiments [214].

#### 5.8.2. Intradermal Capsaicin Model

The principal mechanism of the intradermal capsaicin model involves the direct injection of capsaicin into the dermis. The intradermal injection of capsaicin directly activates the TRPV1 channels on nociceptive neurons, causing a rapid influx of calcium and sodium ions. This process involves the release of bioactive mediators such as calcitonin gene-related peptide (CGRP), neurokinin A, nitric oxide, and prostaglandins [215]. The injection induces a localized, intense pain response followed by a long-lasting area of secondary hyperalgesia and allodynia [215,216]. The initial pain response is followed by a prolonged area of secondary hyperalgesia that is characterized by increased sensitivity to mechanical stimuli. Studies propose various experimental pain models for developing neuropathic pain treatments. However, most research uses these models on healthy people [217]. Due to its ability to induce pain and assess pain medication effectiveness, intradermal capsaicin injection has become a popular and safe method for pain research, allowing scientists to explore the pharmacology of various pain medications [218]. By investigating the mechanisms behind capsaicin-induced hyperalgesia at both nervous system levels, this model holds promise for advancing our knowledge of chronic pain conditions. It causes a transient, sharp pain that is followed by persistently elevated sensitivity (secondary hyperalgesia). This helps evaluate the efficacy of analgesics since it replicates aspects of chronic pain [219]. The crossover design of this method enables reliable comparisons between pain medications’ effectiveness and statistically robust results even with fewer participants. Additionally, capsaicin’s potent and consistent ability to induce pain makes it a preferred choice for pain research [220]. Research has demonstrated the usefulness of intradermal capsaicin injection as a reliable and convenient method for assessing pain and evaluating the efficacy of pain medications in various clinical studies.

While a new capsaicin injection study confirmed the effectiveness of pregabalin for pain, its usefulness for chronic pain research has limitations [221]. Studies show that people with chronic pain, like sciatica, react differently to the model compared to healthy volunteers [222,223]. This suggests that the capsaicin model might not fully capture the mechanisms behind chronic pain, as those mechanisms may not be present in pain-free individuals. The heat/capsaicin model uses a combination of heat and capsaicin to induce sensitization while the intradermal capsaicin model relies solely on the direct injection of capsaicin. Other key differences are the method of sensitization, the pain response, and the duration of sensitization. In conclusion, both models are valuable tools in pain research, each with unique advantages and mechanisms. The choice between them depends on the specific research goals and the aspects of pain being studied. To enhance our comprehension of chronic pain and facilitate the development of more efficacious treatments, it is recommended that future research prioritize the utilization of the capsaicin model in patients who are experiencing real chronic pain conditions.

#### 5.8.3. The Cold Pressor Model

The cold pressor test (CP test), a well-established method for inducing and studying cold pain caused by nerve fiber activation in skin receptors, faces limitations in reliability due to inconsistencies in terms of the equipment used. This model has been widely employed in pain research to investigate pain perception, analgesic effects, and pain modulation mechanisms. It involves immersing a hand or forearm in ice water and measuring pain perception through three main factors: time to initial pain, pain intensity using a visual analogue scale (VAS), and pain tolerance measured by withdrawal time [224]. The immersion of a limb in ice-cold water activates peripheral nociceptors, specifically cold-sensitive nociceptors. These nociceptors are specialized sensory neurons that respond to noxious cold stimuli. The activation of these nociceptors leads to the transmission of pain signals to the central nervous system [211]. Several ion channels are involved in the transduction of cold stimuli. The transient receptor potential melastatin 8 (TRPM8) channel is a primary cold sensor. When activated by cold temperatures, the TRPM8 channels open, allowing the influx of calcium and sodium ions, which depolarizes the neuron and generates action potentials [225,226]. The activation of nociceptors by cold stimuli can trigger neurogenic inflammation. This process involves the release of neuropeptides such as Substance P and calcitonin gene-related peptide (CGRP), which contribute to the inflammatory response and further sensitize the nociceptive neurons. Additionally, other ion channels such as TRPA1 and voltage-gated sodium channels may also contribute to cold-induced pain. This model is valuable for studying pain mechanisms and evaluating the efficacy of analgesic interventions in human volunteers. The primary outcomes measured in this model include the time to onset of pain, pain intensity, and pain tolerance.

The cold pressor pain model, with its controlled approach, enables the analysis of pain responses, the evaluation of pain management interventions, and improved reproducibility using standardized protocols. However, achieving consistent results can be challenging due to variations in equipment. Existing models suggest that Aδ fibers transmit cold sensations, while C fibers likely handle cold pain [227]. Cutaneous vein nociceptors are believed to be responsible for the resulting cold pressor pain. The CP test remains popular due to its established research base, ease of use, affordability, safety, and standardized procedures [228,229,230]. However, genetics play a leading part in pain perception. Variations in specific genes, like the TRPA1 gene, can influence pain sensitivity. For example, the TRPA1 gene variant rs11988795 G>A is linked to heightened cold pain perception [231].

Another large study linked a specific gene (TSSC1) to pain sensitivity using online surveys and at-home cold tests. Interestingly, higher sensitivity was not directly tied to chronic or acute pain but did involve brain development genes and suggested a complex genetic influence on pain perception, including the known link between red hair and MC1R gene variation [232]. Future genetic studies should account for demographic and psychological factors that might influence pain experiences. The cold pressor test can be used to study pain modulation mechanisms, such as the gate control theory of pain. According to this theory, non-painful stimuli can inhibit the transmission of pain signals at the spinal cord level, thereby modulating the pain experience [233]. It is concluded that the cold pressor pain model is a valuable tool in pain research, providing insights into the mechanisms of pain perception and modulation. Its reproducibility and ability to induce a controlled pain response make it an essential model for studying pain and testing analgesic interventions. Despite its limitations, the cold pressor test remains a valuable tool in comprehending the mechanisms of cold pain and studying the impact of genetics on pain perception.

#### 5.8.4. The Ultraviolet Light UV-B Pain Model

Researchers often use UVB radiation to study pain because it is a reliable way to induce inflammation and measure pain sensitivity in both animals and people. This is especially helpful for developing new pain medications, as it allows scientists to see if treatments that work in animals might also benefit humans. When exposed to controlled amounts of UVB light, the skin becomes inflamed, which allows researchers to assess how people perceive different types of pain. Interestingly, when using this model, patients’ sensitivity to heat initially increases but quickly returns to normal. On the other hand, sensitivity to touch and pressure peaks within a few days before subsiding. Notably, during this same period, sensitivity to electrical pain also rises significantly. This suggests that a heightened activity in nerves might be a factor in how people perceive heat pain after UVB exposure [234]. Sunburn pain stems from UVB radiation activating the TRPV4 channels in skin cells. Blocking these channels in mice lessened their pain, tissue damage, and a pain-signaling molecule. Likewise, sunburns in humans elevate TRPV4 levels, making it a promising target for future therapies to combat UVB-induced pain and damage [235].

Recent studies in healthy volunteers provide evidence that the UV-B pain model affects pain perception beyond the directly exposed skin area. This suggests that the model increases sensitivity in nerve cells that respond to pressure or touch, even in surrounding areas. These findings strengthen the case for using the UV-B model in pain research on humans [236]. Scientists often use the UVB pain model to test anti-inflammatory pain meds because it mimics real-world inflammation and responds well to these medications [237]. This model typically involves applying a carefully measured dose of UVB light (either two or three times the amount that causes minimal redness) to a person’s skin. The lower dose (2MED) is preferred for studying inflammatory pain because it creates consistent pain sensitivity without a high risk of darkening the skin (a side effect called post-inflammatory hyperpigmentation). This makes the 2MED UVB model ideal for the early testing of new pain medications, as it balances reliable pain induction with minimal side effects. The UV-B model demonstrates promise for evaluating analgesic drugs and studying sunburn-induced hyperalgesia in human volunteers. A pilot study explored the effects of UV light exposure from tanning beds on chronic pain in fibromyalgia syndrome (FMS). The results suggested a minor decrease in pain scores and an improved mood with UV exposure, indicating the potential for pain relief in FMS [238]. However, significant pain improvements were observed only in one pain assessment measure. Further research is needed to confirm these findings. However, this model’s applicability to studies involving additional pain biomarkers requires further investigation, and alternative models may be needed in such cases [239,240].

### 5.9. Role Human Volunteers in Advancing Pain Research and Ethical Considerations

It is important to prioritize non-animal techniques when conducting research on human pain in order to gain insights into the underlying biology and develop effective treatments. However, advancements in human neuroimaging and human organoids offer potential replacements for certain animal experiments in the study of human pain [205,241]. Recent advancements in pain research, particularly the understanding of pain mechanisms, have been significantly aided by the invaluable participation of human research subjects. By taking part in clinical studies and evaluations, these committed individuals help researchers better comprehend the character of pain, its causes, and potential pain remedies.

Studying individuals with pain, both acute and chronic, helps researchers untangle the complex interplay between biology, psychology, and environment in pain perception. Modern brain imaging tools like fMRI and PET scans have been game-changers, allowing scientists to visualize brain activity during pain processing [242]. Collaborations including the International Pain Genetics Consortium have helped identify genetic variations linked to disorders including a higher sensitivity to pain and chronic pain [243]. These results aid in the development of individualized pain management techniques. Human volunteers are vital for clinical trials that evaluate the safety and effectiveness of novel pain relief treatments. The effectiveness of medications, therapies, and medical devices is evaluated in these studies with the help of critically important volunteers. Recent studies on cutting-edge treatments, such as non-invasive neuromodulation methods, have highlighted the potential of these interventions to reduce pain without relying entirely on pharmaceutical treatments [244]. The intricate interactions between psychosocial factors and pain perception are also better understood with the help of human volunteers. According to another study, psychological factors such as anxiety and sadness can heighten individuals’ sensitivity to pain. The utilization of volunteers in these studies provides valuable insights into the interplay between the mind and body in pain regulation [245].

Researchers must put participants’ health and safety first, making sure that research is carried out by laws and ethical standards [246]. In pain research, human volunteers make incalculable contributions that advance our comprehension of pain’s complexity and allow for the creation of ground-breaking treatments. Volunteers have contributed to the discovery of important insights into pain perception and management through neuroimaging studies, genetic investigations, therapeutic trials, and psychosocial analyses. The commitment of these people remains a crucial cornerstone in the effort to lessen suffering and enhance the quality of life for those who are impacted by pain as pain research develops.

### 5.10. Neuroimaging Techniques in Pain Research

Neuroimaging techniques such as fMRI, PET, and electrophysiological methods have greatly advanced our knowledge of how pain is processed in both humans and animals. Brain imaging techniques like fMRI, PET, and EEG have revolutionized our understanding of pain processing in humans and animals, revealing the complex neural mechanisms behind pain perception. These combined approaches provide a comprehensive picture of the neural networks underlying pain, paving the way for advancements in pain management strategies [242,247,248,249]. Animal models offer several advantages, including experimental control and access to invasive techniques, facilitating detailed investigations of pain-related neural circuits and processes. Animal models provide a critical platform for studying pain, enabling researchers to investigate the neurobiology of pain and explore potential therapeutic interventions. In this article, we have reviewed recent advancements in the neuroimaging of pain research using animal models, highlighting the contribution of various imaging modalities and their applications in understanding pain mechanisms [250]. Furthermore, we discuss key findings from recent studies, shedding light on the neural correlates of pain in animals and providing a foundation for translational research in humans.

#### 5.10.1. Functional Magnetic Resonance Imaging (fMRI)

The use of functional magnetic resonance imaging (fMRI) has brought about a revolutionary shift in our comprehension of pain perception and the impact of pain medications on the human brain. Researchers can explore the neural underpinnings of pain and track the real-time impacts of pain medications using this non-invasive neuroimaging technique. In this article, we explore the application of fMRI in pain drug research, discussing its advantages, limitations, and recent advancements [251]. Furthermore, we emphasize the potential of fMRI to advance the progress of innovative pain treatments. In recent times, fMRI has emerged as a potent tool for investigating pain-related brain activity in animals without the need for invasive procedures [252].

Researchers are increasingly turning to fMRI technology to investigate how pain is processed in the brains of different animals, from rodents to non-human primates. These studies have revealed important insights into the functional organization of pain circuits, cortical plasticity, and the influence of genetic and pharmacological manipulations on pain perception. fMRI has provided unprecedented insights into the neural underpinnings of pain perception and the effects of pain medications. By mapping pain-related brain regions, investigating neuroplasticity, and identifying individual differences in drug responses, fMRI has the potential to transform pain drug research [253]. Although certain limitations exist, ongoing advancements in fMRI techniques hold promise for addressing these challenges and furthering our understanding of pain modulation. Ultimately, fMRI could pave the way for personalized pain medicine and the development of more effective and targeted pain therapies.

#### 5.10.2. Positron Emission Tomography (PET)

PET imaging allows researchers to examine molecular processes associated with pain by utilizing radiotracers that are specific to various neurotransmitter systems. Recent advancements in PET imaging techniques, such as simultaneous PET-MRI, have enhanced the method’s spatial resolution and multimodal imaging capabilities, further enriching pain research in animal models. Positron emission tomography (PET) has demonstrated potential in unraveling the fundamental mechanisms of pain and has made substantial contributions to the field of pain research. This article aims to review recent studies utilizing PET in pain research, highlighting its applications, advancements, and potential future directions. PET allows the visualization of neurotransmitter receptors, transporters, and enzymes involved in pain processing. Recent studies have utilized radiotracers such as [11C]raclopride for dopamine receptors, [11C]PK11195 for neuroinflammation, and [11C]carfentanil for opioid receptors to investigate pain-related neurochemical changes [254]. Recent studies have utilized PET to investigate the role of neurogenic inflammation in pain sensitization [255]. The integration of PET with other imaging techniques like functional magnetic resonance imaging (fMRI) or electroencephalography (EEG) enables the acquisition of complementary data on pain-associated brain functions, leading to a more comprehensive understanding of pain processing. Further advancements in multimodal imaging approaches and targeted radiotracer development hold great promise for enhancing our understanding of pain and improving pain management strategies.

#### 5.10.3. Neuroimaging on Brain Circuitry, Plasticity and Pain Modulation

Neuroimaging investigations conducted on animal models have shed light on the essential brain regions associated with pain processing and modulation. Recent discoveries emphasize the involvement of the anterior cingulate cortex (ACC), insular cortex, thalamus, and periaqueductal gray (PAG) in the processing of pain. Moreover, optogenetic and chemogenetic techniques have enabled the precise modulation of specific neuronal populations, revealing the causal relationships between these regions and pain-related behaviors [256]. Animal models have played a critical role in uncovering the neuroplastic changes linked to chronic pain conditions. By tracking brain changes over time using brain imaging techniques (longitudinal studies), scientists have observed how pain processing areas of the brain are physically altered in terms of their structure and function in chronic pain patients [242]. These findings help us to understand the harmful changes that contribute to chronic pain. Additionally, brain imaging studies in animals have been instrumental in evaluating the effectiveness of various treatments, including medications and nerve stimulation techniques, for alleviating chronic pain.

Brain imaging in animals is improving our understanding of pain, but further work is needed. Future research should focus on better imaging techniques, combining different methods, and creating more realistic animal models of pain. The International Association for the Study of Pain (IASP) has established a task force to propose evidence-based criteria for evaluating the suitability of brain imaging measures in clinical and legal settings [249]. Ultimately, the goal is to translate these findings into effective pain treatments for humans. Further, continued advancements in neuroimaging technologies and innovative experimental designs will pave the way for novel discoveries and therapeutic breakthroughs in pain research. Further advancements in multimodal imaging approaches and targeted radiotracer development hold great promise for enhancing our understanding of pain and improving pain management strategies.

### 5.11. Microbiome in Pain Research

The gut microbiome, consisting of countless microorganisms, has been recognized as a significant contributor to numerous aspects of both health and disease. It has been increasingly recognized that these microorganisms regulate various aspects of human health, including immune function, digestion, and even the nervous system [257]. Recent studies have shed light on the involvement of the gut microbiome in regulating pain sensitivity, presenting a novel area of investigation in pain research. Promising evidence indicates that the makeup of the gut microbiome can impact pain perception and potentially contribute to the onset of chronic pain conditions. Several mechanisms have been suggested to explain the association between pain and the gut microbiome [258,259]. Animal models have been instrumental in elucidating the complex interactions between the gut microbiome and pain perception. This section explores the latest findings and research using animal models that regards the microbiome’s role in pain modulation and discusses potential implications for human health. Evidence indicates that the gut–brain axis plays an important part in the regulation of pain, as there is a reciprocal communication between the gut microbiome and the CNS that impacts pain perception [260]. One study examined the gut microbiota compositions in diabetic neuropathy patients (DNPs) and found significant alterations in the microbial profiles compared to healthy controls. The researchers identified specific bacterial taxa associated with DNPs, highlighting a potential link between the gut microbiome and neuropathic pain in the context of diabetes [261].

Recent research has employed various animal models to examine the impact of the gut microbiome on pain sensitivity, utilizing approaches such as fecal microbiota transplantation (FMT), germ-free animals, and probiotic interventions. For instance, researchers have shown that FMT from pain-resistant mice to pain-sensitive mice can alleviate pain responses, suggesting a direct link between the gut microbiome composition and pain modulation [262,263]. The gut microbiome communicates with the host’s immune system, leading to the secretion of various signaling molecules that impact neuronal activity. This interaction is known to affect pain perception. Research using germ-free mice has revealed that the absence of gut microbiota alters immune responses and increases pain sensitivity [264]. The gut microbiota is a prolific producer of metabolites, encompassing short-chain fatty acids (SCFAs) and signaling molecules like neurotransmitters. These bioactive compounds have the ability to impact neural pathways and thereby influence pain processing in the brain. Recent research suggests that specific microbial metabolites can activate receptors in the gut and the CNS, leading to alterations in pain perception. Additionally, a 2021 study demonstrated that the transplantation of gut microbiota reduced pain in mice with nerve damage, highlighting the potential involvement of the microbiome in the development and maintenance of chronic pain [265]. Another study, published in 2021, showed that gut microbiota transplantation can reduce pain in mice with nerve damage. These findings propose a potential role for the microbiome in both the onset and persistence of chronic pain [266].

More investigation is needed to understand the exact ways in which the microbiome affects pain and to create treatment strategies that focus on the microbiome to effectively manage pain. A study conducted on rats with diarrhea-predominant irritable bowel syndrome explored the impact of a traditional Chinese medicine formula called Tongxieyaofang. The findings indicated that Tongxieyaofang reduced pain by regulating the gut microbiota. The findings suggest a connection between gut microbiota alterations and pain relief in irritable bowel syndrome [267]. Nonetheless, the precise mechanisms underlying the impact of the microbiome on pain remain incompletely understood. However, recent studies utilizing animal models have made substantial strides in enhancing our comprehension of the intricate interactions between the gut microbiome and pain sensitivity. These findings underscore the role of the microbiome in modulating pain responses and provide a promising avenue for potential therapeutic interventions for pain-related conditions in humans. Understanding the mechanisms identified in animal models may provide novel therapeutic targets for managing chronic pain in humans through microbiome interventions.

### 5.12. Computational and Mathematical Models in Pain Research

The computational theory of pain perception is crucial for understanding chronic pain syndromes and optimizing therapeutic programs. However, current pain experiments face challenges like complex pain behaviors and sensation-focused experiences. New theories and models can help overcome these issues with the use of powerful computers and parallel processors to unravel pain’s complexity [268,269] Computational approaches help us identify neural substrates for pain information processing, a key benefit of these approaches. Another mathematical study of pain developed an advanced Bayesian inference model for pain predictions, enabling explicit predictions of impending pain [270,271]. Another key advantage of mathematical modeling lies in its capacity to facilitate intricate models without the need for surgical interventions, rendering it particularly valuable in addressing chronic pain during treatment. Emerging noninvasive mathematical and computational models are now being employed to tackle the multidimensional nature of pain, offering the capability to predict previously unnoticed behaviors associated with pain responses. Incorporating recent neuroanatomic evidence is essential, as existing computer models of peripheral pain currently lack the precision, or at least the accuracy, needed to represent the circuits responsible for processing noxious stimuli, particularly at the spinal level [272,273].

A computation and mathematical study revealed that the nociceptive system generates probabilistic predictions about pain patterns, even without external cues. The study suggests that future research should explore the influence of temporal statistical predictions on pain perception and controllability, which could potentially impact clinical pain management [270]. A recent computational study used a Pavlovian learning task to investigate the impact of prior expectations on pain perception. The findings suggest that individuals who heavily rely on prior expectations during painful experiences may be more susceptible to chronic pain and be more responsive to learning-based interventions. The study compared weighting estimates from the hierarchical Gaussian filter model with established measures of conditioned pain modulation, highlighting the importance of predictive processing in pain perception and the need for personalized models to enhance our understanding of pain [274]. A study replicated and expanded a mathematical model for pain, focusing on enhancing its biological plausibility for broader applications. The model includes two units with inhibitory and excitatory interneurons, T cells, and midbrain cells. Nonetheless, the authors suggested that further refinement is essential to constructing a more comprehensive model that accounts for both acute and chronic pain.

Further refinement is needed to account for acute and chronic pain with multiple neuronal fiber inputs, plasticity, and descending control mechanisms. A group of researchers systematically reviewed mathematical algorithms and computational models used to characterize pain. Most studies primarily employed classification algorithms to differentiate pain and no-pain conditions, with a predominant focus on identifying the presence of pain rather than exploring diagnostic or treatment features [275]. In conclusion, prioritizing the advancement of models that grasp the underlying mechanisms of pain is deemed essential in order to anticipate potential breakthroughs in treatment.

## 6. Animal Models of Pain: A Critical Evaluation of Validity

Animal models are indispensable tools in pain research that allow for advancing our understanding of pain mechanisms and which aid in the development of new analgesics. Animal models of pain are assessed based on their ability to simulate human diseases and accurately predict the outcomes of therapeutic interventions. However, the validity of these models is crucial for translating findings from animals to humans, and it remains a subject of ongoing debate. Despite challenges, we point to recent successes in analgesic drug development that illustrate strategies for increasing the predictive reliability of animal models for pain. Several previous reviews have provided comprehensive and informative summaries of animal pain models and endpoints, considering their validity and reproducibility [276]. Table 1 provides a comparative analysis of different animal models used for pain research, highlighting their advantages, disadvantages, and implications for validity. This section critically evaluates the validity of various animal pain models, focusing on their face, construct, and predictive validity.

Construct validity refers to how an animal model accurately represents the underlying psychological construct or mechanism of human pain. The theoretical soundness of a model is assessed through its construct validity, which determines whether it aligns with relevant theoretical frameworks [277]. Several factors may influence and contribute to the construct validity of a specific animal model of pain. The overlap between the neural pathways activated in animal models and those involved in human pain is crucial. For example, studies have demonstrated similarities in the activation of brain regions such as the anterior cingulate cortex and insula between both humans and animals experiencing pain. Secondly, the behaviour responses exhibited by animals in response to pain stimuli should align with those observed in humans. Interpreting animal behavior can be challenging regardless of the model or measurement used to study pain [278]. The activation of neural pathways involved in pain signaling has physiological, endocrine, and behavioral consequences. This includes nociceptive responses like withdrawal reflexes and avoidance behaviours, as well as affective responses such as distress and reduced activity.

A systematic review and meta-analysis were conducted to assess the behavioral outcomes of Complete Freund’s adjuvant-induced inflammatory pain in the rodent hind paw. The study quantifies CFA’s effects on rodent pain behaviors, providing insights for future research. Factors like age, sex, and species influence these effects. Researchers can use this information to optimize studies and improve our understanding of chronic pain [279]. Finally, the physiological or pathological processes underlying mechanisms of pain in animal models should be comparable to those in humans. Assessing pain in laboratory animals and developing effective analgesics requires a comprehensive understanding of the nociceptive pathway and the neurobiological mechanisms underlying observable changes in facial expressions [280]. This involves considering factors such as peripheral nerve damage, inflammation, and central sensitization. For instance, previous studies have shown that dopamine and opioid systems are involved in the pain associated with neurodegenerative diseases. Preclinical models can provide deeper insights into the cellular and molecular mechanisms involved and aid in the development of more effective analgesics. It remains necessary to enhance animal models’ construct validity by selecting or refining models of pain that accurately reproduce human pain. The formalin nociception test is a widely predictive model that is valid for studying acute pain in rodents and allows researchers to observe and measure pain-related behaviors like itch by exciting sensory neurons through the direct activation of TRPA1. In one study, blocking or genetically removing TRPA1 significantly reduced the flinching, licking, and lifting behaviors observed after injecting formalin into the paw, indicating a key role in formalin-induced pain [57].

Colorectal distension (CRD) is one of the most reliable methods for studying visceral pain in both humans and animals. It accurately simulates the intensity and referred pain experienced by patients. By increasing the complexity of pain assays, we can improve the predictive validity of translational pain treatments and accelerate the process of bringing them to patients. This method helps to assess the efficacy of novel analgesic compounds, the effects of strains or genetic differences, and the influences of physical and psychological stressors on colonic pain sensitivity [65]. An experimental study was conducted to validate the murine CCI model for neuropathic pain behavior. Despite its limitations due to environmental factors, the murine chronic constriction injury model remains a valuable tool for studying neuropathic pain behavior and conducting observational studies. Another study demonstrated that ensuring the predictive validity of the CCI model requires a comprehensive battery of behavioral tests to identify clinically relevant and measurable phenotypes for quantifying chronic neuropathic pain [281]. Rodent models of neuropathic pain serve as tools for investigating the underlying mechanisms of pain associated with peripheral nerve damage and for evaluating the efficacy of novel compounds. Nevertheless, few of these models have been fully characterized, and the validity of many remains questionable. Researchers have developed a new rat model SNL using an oblique lateral approach. This model induces neuropathic pain by compressing and irritating spinal nerve roots, leading to inflammation and pain. The oblique lateral approach SNL model is reliable, reproducible, accessible, and less invasive compared to other models. The persistent pain and hyperalgesia symptoms produced by the SNL model closely resemble those observed in clinical cases of lumbar disc herniation-induced spinal nerve root irritation or compression. In essence, the study suggests that the novel SNL model is a valuable tool for research into neuropathic pain, offering a reliable and clinically relevant approach to studying this condition in rats [282].

Streptozotocin-diabetic neuropathy is a chronic pain condition that occurs when nerve damage results from prolonged hyperglycemia. It is commonly characterized by a burning sensation and tactile allodynia. Proinflammatory cytokines are found to play an important role in the pathogenesis and progression of diabetic neuropathy. Several synthetic and natural analgesic compounds have been investigated in rat models of streptozotocin-induced diabetic neuropathy. These compounds have demonstrated the ability to suppress the overactivation of inflammatory molecules and mediators [283]. Another experimental study validated this diabetic neuropathy model for vulvodynia using both systemic and topical gabapentin. Both formulations demonstrated antivulvodynia effects, supporting the model’s value for studying vulvodynia and developing new pain medications. The findings align with clinical studies that have shown the effectiveness of topical gabapentin for treating vulvodynia [95].

Metastatic bone pain is a significant health problem for patients with advanced cancer and significantly diminishes their quality of life. Understanding of the intricacy of cancer-induced bone pain (CIBP) has been greatly aided by animal models. The significance of the interplay of inflammatory variables, bone metabolism, and neurochemical alterations in CIBP has been underscored by recent investigations. The capacity of these animal models to replicate human CIBP and the calibre of the research conducted using them will be crucial, even though their predictive power for therapeutic outcomes has not yet been thoroughly demonstrated [110]. A new mouse model for metastatic bone pain was created by injecting intra-tibial injections of syngeneic MRMT-1 rat mammary gland carcinoma cells into the tibia. This resulted in bone tumors, pain behaviours (like hypersensitivity to touch, mechanical allodynia, and hyperalgesia), and structural damage. The model offers a valuable tool for studying bone pain and developing treatments [284]. A new animal model for CIBP has been developed by implanting Walker 256 tumor cells into the femur. This model features a simple anatomical structure and minimal tissue damage. The model has been shown to accurately replicate time-dependent tumor growth and pain behaviors, making it a promising tool for future research into the mechanisms and treatment of bone cancer pain [285].

The genetic background of an animal model can significantly impact its expression of pain-related genes and phenotypes. A TRPV1^K710N^ knock-in mouse model of pain was developed using CRISPR/Cas9 technology. This model showed that the K710N variant decreased the capsaicin-induced calcium influx in dorsal root ganglion neurons. The model introduced a human genetic variant into the TRPV1 receptor, which is involved in pain sensation. This model allowed researchers to test a potential pain treatment peptide that targets the K710 region of TRPV1, which is crucial for pain signaling. The avian-like TRPV1^K710N^ mice provide evidence that the K710 site in the C-terminus of mammalian TRPV1 is critical for channel gating and nociception [286]. Clinical genetic research has revealed that a loss of Nav1.7 function results in a total absence of acute pain perception. A mouse model lacking Nav1.7 exhibits a phenotype similar to human pain. The role of Nav1.7 in pain perception is conserved between rodents and humans. The Nav1.7 knockout model suggests potential biomarkers for studying Nav1.7-targeted pain therapeutics. The global deletion of Nav1.7 has a more significant effect on pain than the global deletion of Nav1.3, Nav1.8, and Nav1.9. In summary, the study highlights the advantages of the Nav1.7 knockout model in studying pain perception and its potential as a target for developing new pain treatments. This model could be a potential tool in evaluating acute forms of pain using gene knockout pain models of mice. Mice lacking both Nav1.7 and Nav1.8 exhibit normal levels of neuropathic pain, even though they lack inflammatory pain symptoms and have altered mechanical and thermal acute pain thresholds [135,136]. Gene knockout and knock-in techniques can sometimes lead to unintended alterations in other genes, such as off-targets.

Despite numerous reports and systematic reviews highlighting the shortcomings of many animal models in predicting human pain efficacy, the scarcity of research examining their successful applications and negative outcomes makes it difficult to accurately estimate the specificity of any model. Any animal models of pain used in pain drug discovery tend to be validated with standard analgesics, potentially leading to a bias towards tractable pain mechanisms and the development of novel analgesic compounds. By addressing specific limiting factors and implementing appropriate strategies, researchers can improve the reproducibility and validity of genetic models of pain, leading to more reliable and informative research findings. On the other hand, face validity, reflecting a pain-like state in animals, should be achievable and is of paramount importance. The behaviors exhibited by animals should resemble those observed in human pain. For example, facial expressions, vocalizations, and posture can be assessed for similarities. By considering factors such as the construct, predictive, and face validity factors, researchers can select appropriate models and interpret their findings with greater confidence in conducting pain drug discovery. The combination of distinct animal models may be a solution to the lack of good predictive validity of animal models that basic research is facing so far. As our understanding of pain mechanisms and the limitations of animal models evolves, new and more sophisticated models will likely emerge in the future. Systematic reviews and meta-analyses of animal studies can provide a comprehensive overview of the validity and usefulness of these studies. By combining the results of multiple studies, researchers can gain a clearer understanding of the strengths and limitations of different animal models.

## 7. Progress and Challenges in Translational Pain Research

Studies using animals have been crucial for understanding pain, developing new pain medications, and exploring treatments for chronic pain conditions. These models serve as a vital component of preclinical research, enabling scientists to delve into the intricacies of pain and assess interventions before conducting human trials. Despite limitations that necessitate ongoing refinement to bridge the gap to human applications, animal models remain crucial for pain research, aiding scientists’ understanding of pain mechanisms and the development of novel pain medications. They have also helped identify pro-inflammatory mediators and cytokines involved in pain sensitization.

One of the primary challenges with animal models of pain is their translational relevance to humans. Pain perception and processing may differ between species, making it challenging to extrapolate findings directly from animals to human patients [287,288]. It may be difficult to adequately represent and comprehend the subtleties of pain in animals because such indirect assessments fail to fully capture the holistic pain experience. Factors such as neuroanatomy, neurochemistry, and behavioral responses differ across species, potentially leading to inaccuracies and limited generalization. This discrepancy has led to several instances of promising preclinical results not translating into successful clinical outcomes. Moreover, translating findings from acute to chronic models of pain conditions is complex and may require the design of specialized models to better mimic the long-term nature of human pain. Using animals in pain studies raises ethical concerns regarding animal welfare and potential suffering. Researchers must use prudence and think carefully about the restrictions and moral ramifications of utilizing such models. To reduce the use of animals while maximizing the relevance of the data, researchers must abide by strict ethical norms and actively investigate alternatives, such as in vitro models and computer simulations. Pain is a multi-dimensional experience influenced by psychological, emotional, and social factors [289]. Animal models often focus on isolated aspects of pain, potentially overlooking the full complexity of human pain. Efforts are being made to develop more comprehensive models that incorporate these multifaceted aspects. Assessing pain in animals relies heavily on behavioral responses, which can be subjective and challenging to interpret accurately [290,291]. The development of more objective measures, such as neural imaging or biomarkers, could enhance the reliability and validity of pain assessments in animal models. While animal models have revolutionized pain research, uncovering mechanisms and treatments, limitations hinder their perfect translation to humans. Continued refinement and responsible use are essential to bridging this gap and developing better pain management strategies for patients. Recognizing these limitations and exploring alternative approaches, like human-based research, will ultimately strengthen our fight against chronic pain.

## 8. Pain Models and Analgesic Drug Discovery

The heterogeneity of chronic pain in humans has been a major obstacle in developing more effective pain medications. Decades of pain research have highlighted the difficulty of translating preclinical findings into clinical applications. To accelerate the discovery and development of novel pain medications, more predictive models are urgently needed. Despite extensive preclinical research, the challenge of translating findings into effective chronic pain treatments remains, as evidenced by the limited number of new, safe, and effective drugs [292]. Validating new pain models and endpoints is crucial for drug discovery, which requires molecular data from diverse assays, including different animal models, to understand disease mechanisms and identify novel pain therapeutic targets and drugs. Preclinical pain models often fall short due to their limited ability to replicate the underlying mechanisms of human pain and their reliance on endpoints that may not fully capture the human pain experience [11,293]. Preclinical pain researchers must accurately model complex human pain experiences in animals to better understand its underlying mechanisms. While progress is being made in other areas of chronic pain research, continuous model refinement and validation are essential for overcoming limitations and accurately assessing therapeutic potential. Pain research requires bidirectional translation: adapting animal models to clinical findings and applying animal data to clinical practice. Pain drug discovery and target identification should not be limited to animal models but should also incorporate data from human neuronal cells and single-cell neuronal studies. By combining genetically modified animals with fMRI and human sensory neuron models, we can bridge preclinical and clinical research. The direct connection of fMRI and other imaging techniques to human studies, coupled with the ability of HiPSCs to mimic patient phenotypes, provides a powerful approach for identifying pain targets and predicting drug safety more accurately than traditional animal models [48,171]. This approach will help to identify pain targets by using data from both animal and human models. Technological advancements are driving the development of more refined animal models that better replicate human pain conditions, facilitating the discovery and development of new pain medications by bridging the gap between preclinical research and clinical outcomes.

### Novel Pain Drug Development Using Animal Models

**Figure 7:**
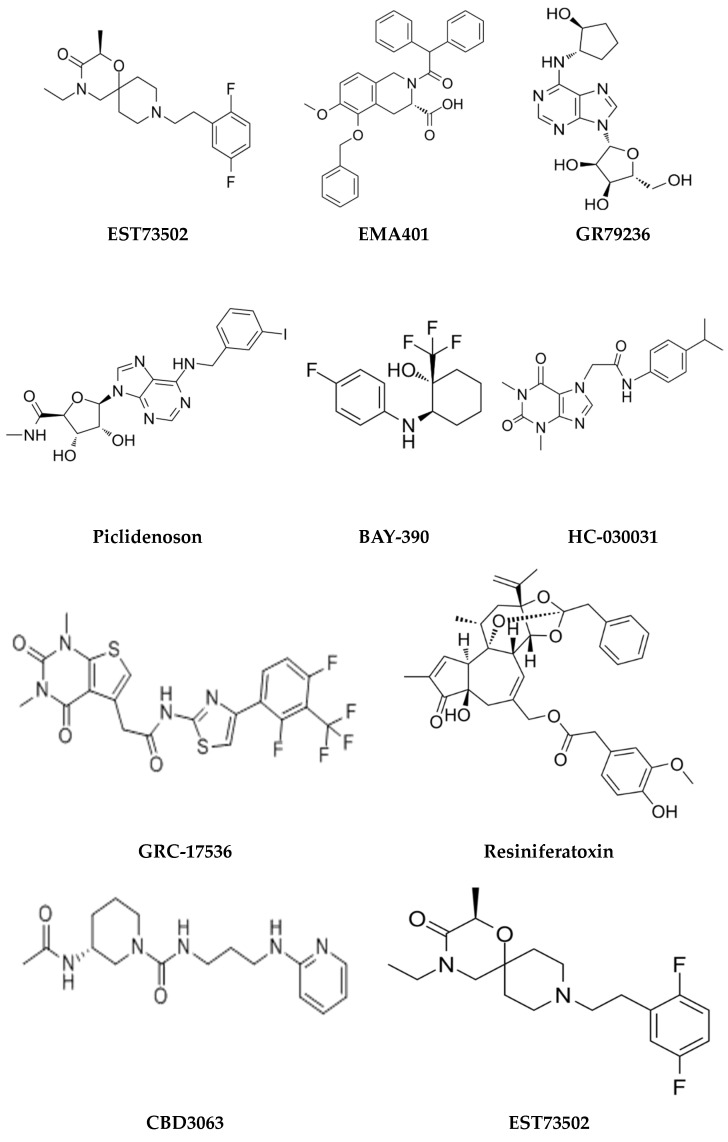

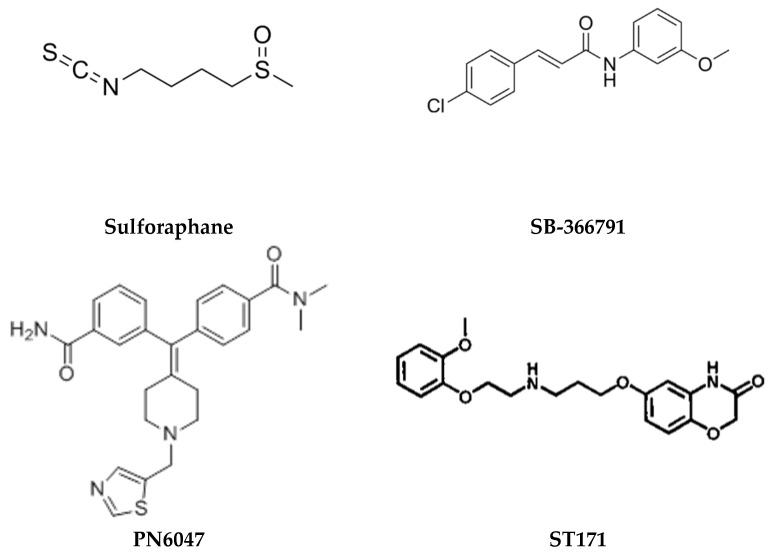
The chemical structure of some novel compounds under pain drug development.

Rodent pain models have been instrumental in advancing our knowledge of pain mechanisms, yet their translation into clinically effective analgesics remains challenging. Despite stringent evaluation, only 57% of novel analgesic candidates successfully transition from phase 3 clinical trials to regulatory approval. Several novel analgesic candidates failed to reach regulatory approval due to issues such as inaccurate data and ineffective trial methodologies [294]. Table 2 and Figure 7 outline some novel analgesic compounds’ efficacies against different animal models, highlighting their targeted mechanisms. EMA401 is a novel, nonopioid analgesic drug being developed for the treatment of peripheral neuropathic pain. EMA401 demonstrated comparable pain-relieving effects to a benzoxazole analogue and pregabalin in CCI rats, as evidenced by its ability to alleviate mechanical allodynia in the ipsilateral hind paws [295,296]. EMA401 effectively prevented the development of acute paclitaxel-induced neuropathic pain in mice, as demonstrated by its ability to inhibit both mechanical and cold allodynia in the hind paws [297]. Despite promising preclinical results, the clinical development of EMA401 was halted due to unexpected liver toxicity observed in cynomolgus monkeys during a long-term toxicity study [298]. However, despite early successes, no adenosine-based drugs have yet been approved for pain treatment. Similarly, various selective NaV1.7 channel inhibitors have been developed, but their effectiveness in alleviating pain has been limited in both preclinical models and human clinical trials [299]. Pn3a, a peptide derived from tarantula venom, demonstrated a potent inhibition of NaV1.7 channels while exhibiting high selectivity over other NaV channels. Despite its promising preclinical activity, Pn3a’s in vivo analgesic efficacy has been inconsistent, potentially due to factors such as differences in perineurial permeability between species. Pn3a, a selective NaV1.7 channel inhibitor, serves as a valuable pharmacological tool for investigating the role of NaV1.7 inhibition in pain pathways.

BAY-390, a TRPA1 antagonist, effectively inhibited cinnamaldehyde-induced nocifensive responses in rats in a dose-dependent manner. The observed in vivo efficacy of this drug correlated with plasma concentrations, suggesting that reaching the in vitro IC_50_ is crucial for optimal peripheral TRPA1 inhibition. The TRPA1 agonist CA induces a robust nocifensive response in rats, providing a valuable animal model to study TRPA1-mediated pain and evaluate the efficacy of TRPA1 antagonists. This inhibitor further demonstrated dose-dependent efficacy in alleviating CFA-induced mechanical hyperalgesia in rats, with significant pain relief observed at higher dosages. A re-evaluation of BAY-390 in a neuropathic pain model revealed a delayed yet persistent reversal of mechanical allodynia at a high dose, while a lower dose proved ineffective. These findings suggest that TRPA1 inhibition within the central nervous system is essential to obtain the analgesic effects of BAY-390 on neuropathic pain, underscoring the role of TRPA1 in central pain pathways [300]. Sigma receptors, particularly sigma-1 receptors, offer promising targets for drug development in pain management due to their abundance in the endoplasmic reticulum [301,302]. EST73502, a novel dual µ-opioid receptor agonist/σ1 receptor antagonist, is currently undergoing clinical trials for the treatment of osteoarthritis-related pain [303]. Compound 14u (EST73502) exhibited potent analgesic effects in preclinical pain models, comparable to oxycodone, due to its dual activity as a µ-opioid receptor agonist and σ1 receptor antagonist. ADV-502, a novel bifunctional compound targeting both µ-opioid receptors and sigma-1 receptors, has shown promising preclinical efficacy in various pain models, including acute and visceral pain, suggesting potential benefits beyond those of traditional opioid monotherapy [304]. ADV502, a novel bifunctional compound, holds the potential for both pain management and opioid rehabilitation. The bifunctional compound ADV-502, combining µ-opioid receptor agonism with σ1 receptor antagonism, offers a promising approach for developing more potent and safer analgesics.

The NGF-antagonism study was investigated using a humanized anti-NGF antibody, PG110, on established chronic pain models in rats. PG110 effectively attenuated chronic inflammatory pain but showed limited efficacy in neuropathic pain, suggesting that targeting NGF may be a promising therapeutic strategy for certain types of persistent pain [305]. This study investigated the effects of NGF-antagonism using a humanized anti-NGF antibody, PG110, on an established Freund’s adjuvant (CFA) rat model of persistent inflammatory pain and the L5 spinal nerve axotomy (SNA) model of peripheral neuropathic chronic pain models in rats. PG110 effectively attenuated chronic inflammatory pain but showed limited longevity. PG110 effectively attenuated chronic inflammatory pain but showed limited efficacy in neuropathic pain, suggesting that targeting NGF may be a promising therapeutic strategy for certain types of persistent pain [306]. Targeting the Nrf2 pathway has shown promise in analgesic therapy, with studies demonstrating the effectiveness of compounds like sulforaphane in alleviating pain. Nrf2, a transcription factor, plays a crucial role in regulating cellular redox status and has anti-inflammatory properties, contributing to pain management [307]. Tanezumab, a humanized monoclonal antibody targeting NGF, is being investigated in clinical trials for various pain conditions, representing a promising therapeutic approach for NGF-mediated pain. It initially showed promise in preclinical and early-stage clinical trials for pain management, particularly in patients who are resistant to traditional analgesics [308]. However, in 2010, the FDA halted clinical trials of tanezumab due to safety concerns.

Sulforaphane, when administered alone or in combination with SNC-80 (0.5 mg/kg), a δ-opioid receptor agonist, could be a potential therapeutic approach for painful diabetic neuropathy in db/db mice associated with type 2 diabetes [309]. Sulforaphane (SFN) dose-dependently alleviated pain hypersensitivity in a chronic constriction injury (CCI) model, and was associated with a reduction in pro-inflammatory cytokines (TNF-α, IL-1β, IL-6) and an increase in the anti-inflammatory cytokine IL-10 [310]. Adenosine receptors, particularly A1 and A3, have demonstrated promising potential for treating neuropathic pain, with evidence supporting their effectiveness across preclinical, experimental, and clinical settings. Novel drug modalities like RNA therapeutics and cell therapies offer promising avenues for developing new adenosine-based pain treatments. Preclinical and clinical studies have highlighted the potential of targeting adenosine receptors for pain management, with A1R agonists (GR79236) showing promise in headache disorders and A3R (IB-MECA) agonists demonstrating efficacy in mice models of neuropathic pain associated with chemotherapy [311]. These neuropathic animal models support the therapeutic potential of adenosine modulation in neuropathic pain, provided that selective and safe drugs can be developed.

TRP channels have emerged as a promising target for pharmacological intervention in pain management. HC-030031, a TRPA1 antagonist, demonstrated efficacy in alleviating mechanical hypersensitivity in both inflammatory and neuropathic pain models in rodents [125,312]. Topical TRPA1 antagonists, such as HC-030031, may have therapeutic value for treating pain associated with conditions like sunburn or thermal injury. A topical gel containing 0.05% HC-030031 effectively reversed both mechanical and cold allodynia in mice following ultraviolet B-induced burn injury [313]. GRC17536, another TRPA1 antagonist, showed promise in reducing pain scores in patients with painful diabetic polyneuropathy, highlighting the potential clinical benefits of targeting TRPA1 for pain management [314]. The intraplantar injection of resiniferatoxin (RTX), a TRPV1 receptor agonist, effectively reversed and prevented the development of neuropathic pain in L5 nerve injury rats, demonstrating its potential as a therapeutic agent for chronic pain conditions. The analgesic effects of RTX were associated with changes in gene expression and immunohistochemical markers in the spinal cord and dorsal root ganglia, suggesting a complex mechanism of action involving the modulation of nociceptive pathways. The combination of this TRPV1 antagonist and morphine has potent analgesic effects on bone cancer pain in mice. This was demonstrated in a study using C3H/HeJ mice injected with osteolytic sarcoma cells [315].

The TRPV1 antagonist SB366791, at doses of 0.3 and 1.0 mg/kg, significantly reduced spontaneous flinches in mice with bone cancer pain. When combined with morphine, the analgesic effects of SB366791 were further enhanced, suggesting a synergistic interaction between the two drugs [316]. Additionally, SB-366791 has demonstrated analgesic effects in rats with dental pain and has been shown to prevent alveolar bone loss in a rat model of periodontal disease [317]. CBD3063, a novel compound targeting Cav2.2 calcium channels, demonstrated potent antinociceptive effects in a spared nerve injury (SNI) rat model of neuropathic pain, effectively reducing both mechanical and cold allodynia. The efficacy of CBD3063 in alleviating neuropathic pain was comparable to that of gabapentin, a widely used first-line analgesic for this condition [318]. Several drug candidates targeting TRP and Cav channels are currently being evaluated in mouse models of neuropathic pain in preclinical trials.

A novel bifunctional peptide, KGNOP1, was developed to address the limitations of existing pain medications by combining both antinociceptive and anti-neuropathic pain properties. KGNOP1 demonstrated superior efficacy and safety compared to traditional opioids like tramadol and morphine in treating both neuropathic and nociceptive pain in rats (chronic constriction injury of the sciatic nerve, hot and cold plate tests). These animal models support the potential of KGNOP1 as a promising candidate for the dual treatment of nociceptive and neuropathic pain, making it an ideal choice for future clinical development [319]. PN6047, a novel δ-opioid receptor agonist, demonstrates a biased signaling profile, engaging G-protein signaling while exhibiting partial activity in arrestin-mediated pathways. PN6047 exhibits promising analgesic properties in various preclinical models (sciatic nerve ligation, mono-iodoacetate–induced osteoarthritic pain model, and carrageenan-induced acute inflammation and inflammatory pain) without inducing seizures or respiratory depression, suggesting its potential as a safer and more effective δ-opioid receptor agonist [320].

ST171, a novel 5-HT1AR agonist, demonstrated efficacy in both acute and chronic pain models, suggesting its potential as a therapeutic agent for a range of pain conditions. ST171 exhibited a wider therapeutic window compared to befiradol, highlighting its potential for safer and more effective pain management [321]. Another study investigated the combined effects of NK1 and CGRP antagonists in treating sciatic nerve ligation pain models, demonstrating that their co-administration provided greater pain relief and improved anxiety compared to single-drug therapies. The results suggest that a combination therapy targeting multiple neuropeptide pathways could offer a more effective and well-rounded approach to managing neuropathic and inflammatory pain [322]. These animal models of pain are instrumental in advancing our understanding of pain mechanisms at the molecular level, paving the way for the development of innovative pain drugs, as discussed above. Despite the value of these animal models, developing effective drugs targeting novel pain receptors remains a significant challenge due to the complexity of the molecular mechanisms involved in pain pathways.

## 9. Review Summary

Animal models remain indispensable in pain research, offering valuable insights into pain mechanisms and facilitating the discovery of new analgesics. However, addressing ethical concerns, species differences, and the complexity of pain is crucial for improving the translational success of these models. The need for better pain treatments has led to an increase in animal models, but accurately capturing the complexity of human pain in these models remains a challenge. While they provide valuable insights, they may not fully capture the complexity of human pain. Researchers are exploring alternative models, including genetics, cellular, and humanized models, to address these limitations.

Key areas of focus include:
Molecular pain research: This includes investigating pain at the molecular level using techniques like genomics and proteomics, including employing gene editing, omics, and imaging to enhance pain research. It also includes utilizing high-resolution imaging to study pain pathways in real-time and at a cellular resolution, as well as studying the role of ion channel modulation in pain neurons for potential therapeutic targets.
Genetically Modified Models: Advances in genetic engineering have led to the development of transgenic and knockout mice, allowing researchers to study the role of specific genes in pain pathways.
In vitro models: These involve utilizing human sensory neurons (such as HiPSCs) and stem cell-derived nerves for preclinical research.
Behavioral Assessments: These allow for improved methods for assessing pain-related behaviours in animals, including non-reflexive and voluntary behaviors, to better capture the complexity of pain.
Bridging the gap: This entails addressing the disparity between animal and human pain through refined methods and measures, including developing models that reflect the genetic and phenotypic diversity of human populations, to better understand individual differences in pain perception and treatment responses
Multimodal Approaches: These entail combining different types of pain models (e.g., inflammatory, neuropathic) to study the interplay between various pain mechanisms, as well as combining animal models with genomics, proteomics, and metabolomics to identify novel pain biomarkers and therapeutic targets.

## Figures and Tables

**Table 1:** Overview of Pain Models—Names, Applications, Limitations, and Underlying Mechanisms.

Pain Models		Applications	Limitations	Possible Underlying Mechanisms and Pathways
Inflammatory Pain	Complete Freund’s Adjuvant (CFA)	Pain Mechanism studies and drug screening Neuroinflammation research Behavioral studies	Variability in response based on species difference	Toll-Like Receptors (TLRs): TLR4, Cannabinoid Receptors (CB2R) TRPV1 and TRPA1 channels NLRP3 Inflammasome MAPK Pathway
	Formalin tests	to study both acute and chronic pain phases and evaluate analgesic drugs	It may not fully replicate all types of clinical pain conditions.	Activation of TRPA1 and Pain mediators
Visceral Pain	Colorectal Distension (CRD)	Visceral Pain Research Pain Drug Efficacy Testing Neurobiological studies	Invasiveness	Key receptors involved include TRPV1 and TRPA1 Genetic alterations in neuroplasticity Release of inflammatory mediators
Neuropathic Pain	The Chronic Constriction Injury (CCI)	To study neuropathic pain and test potential treatments for peripheral neuropathy. Behavioural studies Neuropsychiatric disorders	Technically challenge, may not fully replicate all aspects of human neuropathic pain. Variability in response Invasiveness	Increased expression of Nav1.3, Nav1.7, and Nav1.8 channels in (DRG) TRPV1 and TRPA1 MAPK pathway Release of CCL2 and CXCL1
	Spinal Nerve Ligation Model	Neuropathic pain research and Drug screening	Model complexity Species differences	N-methyl-D-aspartate (NMDA) receptors Proinflammatory cytokines (TNF-α, IL-1β, and IL-6) MAPK pathway
	Chronic Constriction of the Infraorbital Nerve Model	Trigeminal neuropathic pain research and drug development Behavioral studies	Variability in response	STAT3 pathways in astrocytes via IL6 TRPV channels
	The Chronic Compression of Dorsal Root Ganglion (CCD)	Drug Development and Neuropathic Pain Research	Variability in response	CXCL12/CXCR4 Signaling
	STZ-induced DNP model	To study mechanisms of painful diabetic neuropathy and to evaluate potential therapies	Various differences in how the model works and how real diabetes happens compared to human diabetes	Increased expression of Nav1.3, Nav1.6, and Nav1.9 channels in DRG neurons
Cancer Pain	Syngeneic Tumor Implantation Bone Metastasis Model Colorectal Carcinoma Metastasis Model	Cancer pain research Immunotherapy studies Tumor-immune interactions Pain drug screening Tumor-microenvironment studies Bone-remodeling studies	Translational relevance Tumor heterogeneity Immune response variability Complexity of pain	Checkpoint Inhibitors: PD-1, PD-L1, and CTLA-4 pathways Dysregulation of RANKL/RANK/OPG system
Genetically Modified	TRPV1 Knockout Mice OPRM1 Knockout Mice TRPV1 Overexpression Mice Nav1.7 Knock-In Mice P2X3 Knockout Mice	Analgesic drug screening Pain Mechanism studies To study the role of *TRPV1* in pain perception To study the role of μ-opioid receptors (MORs) in pain and addiction Pain sensitization studies To study the role of Nav1.7 To study the role of P2X3 receptors	Compensatory mechanism Species differences Opioid Research Behavioral research Overexpression of artefacts	TRPV1 cation channel activation Modulation of MOR (GPCR) pathways Overexpression of TRPV1 Activation of Nav1.7 P2X3 activation
Cellular Models	Human and Rat hDRG Neuronal Cultures Programmed fibroblasts	Pain Mechanism Drug Screening Electrophysiological studies Regenerative medicine studies	Cell viability (The cells may not fully replicate the in vivo environment.	Voltage-Gated Sodium Channels (Nav)

**Table 2:** Examples of novel analgesic drugs under development using various animal models.

Compound Name	Target/Class of Drug	The Type of Pain Studied	The Type of Animal Model Used
EMA401 (Olodanrigan)	Selective AT2R receptor antagonist	Neuropathic Pain	Chronic constriction injury
GR79236 IB-MECA (Piclidenoson)	A1R agonist A3R agonist	Neuropathic Pain	Neuropathic pain models
Pn3a	Nav1.7 antagonist	Nociception Neuropathic Pain Inflammatory pain	Hotplate, formalin, carrageenan, CFA
BAY-390	TRPA1 Agonist	Neuropathic Pain	Cinnamaldehyde
HC-030031	TRPA1 Agonist	Neuropathic pain Inflammatory pain	UV-light B-induced burn injury model (Sun-burn thermal injury)
GRCI7536	TRPA1 Agonist	Diabetic neuropathic pain	Neuropathic pain model
Resiniferatoxin (RTX)	TRPV1 Agonist	Neuropathic pain	L5 Nerve Injury model
CBD3063	Cav2.2 antagonist	Neuopathic pan Inflammatory pain	The spinal nerve injury model, Hot plate, cold plate tests, Chronic constriction injury
EST73502 (Bifunctional ligand)	MOR (µ) agonist Sigma (σ) Receptor antagonist	Neuropathic Pain Cancer Pain Arthritic pain	Partial sciatic nerve ligation Paw Pressure test
Sulforaphane	MOR agonist	Neuropathic Pain	Chronic constriction injury Chronic constriction injury
SB366791 PN6047	VR1/TRPV1 antagonist MOR agonist	Neuropathic Pain Cancer Pain Inflammatory pain Acute dental Pain	The spinal nerve ligation; L5 Nerve injury model Rat model of Periodontal, mono-iodoacetate–-induced osteoarthritic pain model and carrageenan-induced acute inflammatory model
ST171	5HT1AR agonist	Neuropathic pain (Acute and Chronic Pain)	Hot plate, Tail flick, CFA, Spinal Nerve injury models
PG-110 (Antibody)	Anti-NGF	Inflammatory pain	CFA, L5 spinal nerve axotomy (SNA) model
Tanezumab (Antibody)	Anti-Nrf2	Neuropathic pain	Diabetic neuropathic models

Nrf2—nuclear factor erythroid 2-related factor 2; CFA—complete Freund’s adjuvant; Cav2.2—the N-type voltage-gated calcium channel; NGF—nerve growth factor; MOR—mu (µ) opioid receptor; T TRPV1—the transient receptor potential cation channel subfamily V member 1; TRPA1—transient receptor potential cation channel A 1; Nav1.7—the voltage-gated sodium channel isoform 1.7; AR—adenosine receptor; AT—angiotensin receptor II; L5 injury—lumbar 5 injury; VR—vanilloid receptor.
